# Supplementary material for: Mathematical properties of optimal fluxes in cellular reaction networks at balanced growth
Source: PLoS Comput Biol. 2023 Jun 6;19(6):e1011156. doi: 10.1371/journal.pcbi.1011156 (PMC10275479; doi:10.1371/journal.pcbi.1011156)
Supplement: S1 Text — (PDF) [file pcbi.1011156.s001.pdf]

Supporting Information for

Mathematical properties of optimal fluxes in cellular reaction networks at balanced growth

Hugo Dourado, Wolfram Liebermeister, Oliver Ebenhöh, Martin J. Lercher

Hugo Dourado.
E-mail: hugo.dourado@hhu.de

This PDF file includes:

S1 Text
Fig. A
Table A
SI References

S1 Text

1. Detailed derivation of the balance equations

The equations below will be simplified by adopting the Einstein summation convention: a repeated lower and upper index denotes a summation over this index (often indicating a matrix multiplication). Let us consider a Lagrangian including inequality constraints on metabolite concentrations,

[1]

The necessary KKT conditions are the same as in the main text, plus extra conditions for the constraints on metabolite concentrations, including

$$M^m_j\, f^j \geq 0 \quad \text{(primal feasibility)} \tag{2}$$

$$\sigma^m M^m_j\, f^j = 0 \quad \text{(complementary slackness)} \tag{3}$$

The last equation corresponds to $\sigma^m b^m = 0$, but since cells in optimal states will only express those proteins that are actually needed to catalyze reactions, we can be even more restrictive and require a stronger version of the last equation:

$$\sigma^m M^m_j\, f_j = 0 \quad . \tag{4}$$

This equation encodes the following: if $\sigma^m \neq 0$, the metabolite m is inactive ($c^m = 0$), and then all of the reactions j connected to it (j such that $M^m_j \neq 0$) must also be inactive ($f_j = 0$). We also note that because the turnover times $\boldsymbol{\tau}$ differ from zero, the complementary slackness of reaction j (Eq. 50 in the main text) is equivalent to

$$\theta_j f_j = 0 \quad . \tag{5}$$

Now we solve the necessary equality conditions, first considering the stationarity

$$\partial_j \mathcal{L} = \partial_j \mu + \lambda \gamma_j + \theta_l\, f^l\, E^j_j + \theta_j\, \tau_j + \sigma_m\, M^m_j = 0 \quad . \tag{6}$$

By considering the stronger version of the complementary slackness for reactions (Eq. 5), we cancel the sum on θ_l, resulting in

$$\partial_j \mu + \lambda \gamma_j + \theta_j\, \tau_j + \sigma_m\, M^m_j = 0 \quad . \tag{7}$$

Now we multiply (element-wise) by f_j

$$(\partial_j \mu)\, f_j + \lambda \gamma_j\, f_j + \theta_j\, \tau_j\, f_j + \sigma_m\, M^m_j\, f_j = 0 \tag{8}$$

and consider the stronger version of the complementary slackness (Eq. 4) on metabolites, and the complementary slackness on reactions (Eq. 50 in the main text), resulting in Eq. 52 in the main text. Summing over all j and using the density constraint (Eq. 18 in the main text) results in Eq. 53 in the main text. Now the complete expression for λ in terms of $\mathbf{f}$ is given by substituting the expression for the growth rate derivatives (Eq. 31 in the main text),

$$\lambda = -\frac{\mu}{b^{\mathrm{a}}} \left(M^{\mathrm{a}}_j\, f^j - \mu\, \tau_j\, f^j - \mu\, f_l\, E^l_j\, f^j \right) \quad . \tag{9}$$

The first term in parentheses equals b^{a} according to Eq. 15 in the main text, and the third equals $-b^{\mathrm{a}}$ according to Eq. 14 in the main text, so that both terms cancel each other. This results exactly in Eq. 28 in the main text.

Finally, combining Eqs. 52, 28 in the main text, we obtain a general form for the balance equations,

$$\left(\partial_j \mu - \frac{\mu^2}{b^{\mathrm{a}}}\, f_l\, E^l_h\, f^h \gamma_j \right) f_j = 0 \quad , \tag{10}$$

where both indices l and h are used to indicate summation over all reactions. Using Eq. 27 in the main text for the derivative, we have

$$\left(M^{\mathrm{a}}_j - \mu \tau_j - \mu\, f_l\, E^l_j + \mu\, f_l\, E^l_h\, f^h \gamma_j \right) f_j = 0 \quad . \tag{11}$$

We use Eqs. 9,10,11 in the main text to express this in terms of $\mathbf{v}$, $\mathbf{c}$,

$$\left(M^{\mathrm{a}}_j - \mu \tau_j - v_l\, \frac{\partial \tau^l}{\partial c^i} M^i_j + v_l\, \frac{\partial \tau^l}{\partial c^i} \frac{c^i}{\rho} \gamma_j \right) v_j = 0 \quad , \tag{12}$$

Using $v_l = p_l / \tau_l$ from Eq. 4 in the main text, we obtain

$$\left(M^{\mathrm{a}}_j - \mu \tau_j - \frac{p_l}{\tau_l} \frac{\partial \tau^l}{\partial c^i} M^i_j + \frac{p_l}{\tau_l} \frac{\partial \tau^l}{\partial c^i} \frac{c^i}{\rho} \gamma_j \right) v_j = 0 \quad . \tag{13}$$

By multiplying out v_j, we get Eq. 29 in the main text.

2. Rate laws and kinetic parameters

For simplicity, it may be convenient to assume that each component τ^j in the vector of turnover times $\boldsymbol{\tau}$ has a general functional form that depends only on a set $\mathbb{K}$ of kinetic parameters relating reactions j with metabolites m and external reactants n. The simplest general rate law would be the irreversible Michaelis-Menten kinetics, which for a reaction l determines

$$\tau^l(\mathbf{c},\mathbf{x}) = \frac{1}{k^l(\mathbf{c},\mathbf{x})} = \frac{1}{k^l_{\text{cat}}} \prod_m \left(1 + \frac{K^l_m}{c_m}\right) \prod_n \left(1 + \frac{K^l_n}{x_n}\right) \quad , \tag{14}$$

where the kinetic parameters are the corresponding turnover number k^l_{cat}, and Michaelis constants K^l_m for metabolites m, and K^l_n for external reactants n. We may consider that all metabolites m and external reactants n that are not substrates in reaction l have corresponding Michaelis constants equal to zero, so the above equation doesn't depend on their respective concentrations. Note only transport reactions s depend on external concentrations $\mathbf{x}$, so $K^{\text{e}}_n = 0$ for all e, n, and $K^{\text{r}}_n = 0$ for all n.

In that case of all reactions following the irreversible Michaelis-Menten kinetics (14), the (direct) elasticity ε^l_m with respect to a metabolite m is

$$\varepsilon^l_m = \frac{\partial \tau^l}{\partial b^m} = \rho \frac{\partial \tau^l}{\partial c^m} = - \prod_{m' \neq m} \left(1 + \frac{K^l_{m'}}{c_{m'}}\right) \prod_n \left(1 + \frac{K^l_n}{x_n}\right) \frac{\rho \, K^l_m}{k^l_{\text{cat}} (c_m)^2} \quad , \tag{15}$$

where m' are metabolites different than m. The corresponding indirect elasticity E^l_j with respect to a reaction j is

$$E^l_j = \frac{\partial \tau^l}{\partial f^j} = \varepsilon^l_m M^m_j = - \prod_{m' \neq m} \left(1 + \frac{K^l_{m'}}{c_{m'}}\right) \prod_n \left(1 + \frac{K^l_n}{x_n}\right) \frac{\rho \, K^l_m}{k^l_{\text{cat}} (c_m)^2} M^m_j \quad . \tag{16}$$

where we note $\varepsilon^l_i \, M^i_j = \varepsilon^l_m \, M^m_j$ since here $\varepsilon^l_{\text{a}} = 0$ (the Michaelis-Menten kinetics (14) doesn't depend on the total protein concentration c^{a}). When $j = e$ is an enzymatic reaction, the scaling of E^l_e by the respective $-f^e$ and τ^l provides the corresponding control coefficient (Eq. 41 in the main text)

$$C^l_e = -\frac{f_e}{\tau^l} \frac{\partial \tau^l}{\partial f^e} = -f_e \, k^l_{\text{cat}} \left(1 + \frac{K^l_m}{c_m}\right)^{-1} \frac{\rho \, K^l_m}{k^l_{\text{cat}} (c_m)^2} M^m_e = -f_e \, \frac{\rho \, K^l_m}{c_m (c_m + K^l_m)} \, M^m_e \quad . \tag{17}$$

Note that in this case the control coefficients don't depend on the turnover numbers k_{cat}, only on Michaelis constants of metabolites K_m.

A more realistic example would be some generalized kinetics such as the "convenience kinetics" proposed in Ref. (1), which may also depend on other parameters such as Hill coefficients, inhibitor constants, and stoichiometric coefficients, so these may also be necessary to determine the set of kinetic parameters $\mathbb{K}$, and by consequence, the model uniquely. With known rate laws, a model is also uniquely determined by the corresponding triple $(\mathbf{M}, \mathbb{K}, \rho)$.

3. Mass balance and the stoichiometric matrix S

For a stoichiometric matrix $\mathbf{S}$ including all reactions and reactants (internal and external ones), and the corresponding vector $\boldsymbol{w}$ of molecular masses (also known as molecular weights) of reactants, mass conservation within reactions implies

$$\boldsymbol{w}^\top \mathbf{S} = \mathbf{0}^\top \quad . \tag{18}$$

Note that, therefore, the vector of molecular masses must be in the left-null space of $\mathbf{S}$.

If we restrict the stoichiometric matrix to internal reactants i, then the internal product of $\boldsymbol{w}^\top$ with the columns of this new matrix with entries S^i_j is nonzero only for transporters

$$\begin{aligned} w_i \, S^i_s &\neq 0 \\ w_i \, S^i_e &= 0 \\ w_i \, S^i_{\text{r}} &= 0 \quad . \end{aligned} \tag{19}$$

Given that $\mathbf{M}$ is a mass-scaled version of $\mathbf{S}$, these relations are equivalent to Eq. 2 in the main text; only transporters are capable of increasing or decreasing mass within the model.

We note that our considerations about mass conservation presuppose that all reactants also appear explicitly in the model (i.e. they have a corresponding row in $\mathbf{S}$). If some reactants (e.g. water or protons) are omitted from the model for convenience, mass balance is not satisfied. In fact, many models in the literature do ignore some reactants, in particular water; this needs attention in realistic models where water is not only a medium but also a reactant in many biochemical reactions.

4. Examples of GBA models and R code for numerical optimization

We present here 4 simple examples of GBA models. We assume for all models a simple irreversible Michaelis-Menten kinetics (14), so in each model turnover times $\boldsymbol{\tau}$ are uniquely defined by a matrix $\mathbf{K}$ of Michaelis constants and a vector $\mathbf{k}_{\mathrm{cat}}$ of turnover numbers for each reaction. Each row of $\mathbf{K}$ corresponds to a reactant, and each column to a reaction, matching the order in the matrix $\mathbf{M}$ (the entries for external reactants are separated by a horizontal line). See the corresponding ".ods" files for a more detailed description of the models, including labels for reactions and reactants and different growth conditions (i.e. external concentrations) for testing the model. Numerical simulations are done by running the R code in the file "GBA.R", with the variable "modelname" set to the desired model (e.g. modelname $\leftarrow$ "A"). Here $\mathbf{K}$ and ρ are in units of g/L, and k_{cat} in units of h^{-1} (resulting from product mass per protein mass per h). The code exports the results as a corresponding pdf file with relevant plots for visualization, and a csv file with the numerical values for the optimal cell state at different growth conditions.

Figure (A) presents the schematics and the corresponding parameters $(\mathbf{M}, \mathbf{K}, \mathbf{k}_{\mathrm{cat}}, \rho)$ of each model. Metabolites are indicated with circles, and total protein with a rounded square. The numbers labeling reactants and reactions match the corresponding order of rows and columns in $\mathbf{M}$, with the last row corresponding to total protein and last column the ribosome reaction by default. All parameters are arbitrary, with the exception of the ribosome reaction where we use $k_{\mathrm{cat}}^{\mathrm{r}} = 4.55$ and $K_m^{\mathrm{r}} = 8.3$ for its main substrate, based on the estimations for *E. coli* in Ref.(2), and $\rho = 340$ based on the measured *E. coli* dry mass density (3).

Models A and B have the simplest model structures (a linear pathway) for two and three reactions, respectively. Model C has a second transport reaction excreting metabolite "2". Model "D" has two redundant reactions ("3" and "4"), of which only one is active at optimal growth (see optimization results).

A

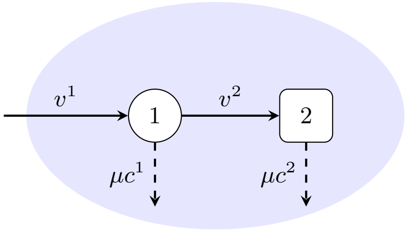

$$\mathbf{M} = \begin{bmatrix} 1 & -1 \\ 0 & 1 \end{bmatrix}, \mathbf{K} = \left[\begin{array}{cc} 1 & 0 \\ \hline 0 & 8.3 \\ 0 & 0 \end{array}\right], \mathbf{k}_{\text{cat}} = \begin{bmatrix} 10 \\ 4.55 \end{bmatrix}, \rho = 340$$

B

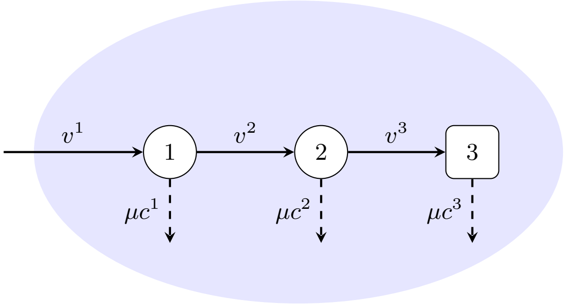

$$\mathbf{M} = \begin{bmatrix} 1 & -1 & 0 \\ 0 & 1 & -1 \\ 0 & 0 & 1 \end{bmatrix}, \mathbf{K} = \left[\begin{array}{ccc} 10 & 0 & 0 \\ \hline 0 & 10 & 0 \\ 0 & 0 & 8.3 \\ 0 & 0 & 0 \end{array}\right], \mathbf{k}_{\text{cat}} = \begin{bmatrix} 10 \\ 8 \\ 4.55 \end{bmatrix}, \rho = 340$$

C

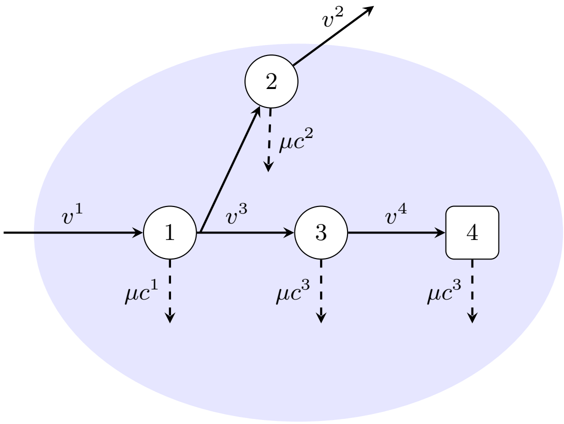

$$\mathbf{M} = \begin{bmatrix} 1 & 0 & -1 & 0 \\ 0 & -1 & 0.2 & 0 \\ 0 & 0 & 0.8 & -1 \\ 0 & 0 & 0 & 1 \end{bmatrix}, \mathbf{K} = \left[\begin{array}{cccc} 10 & 0 & 0 & 0 \\ 0 & 0 & 0 & 0 \\ \hline 0 & 0 & 10 & 0 \\ 0 & 10 & 0 & 0 \\ 0 & 0 & 0 & 8.3 \\ 0 & 0 & 0 & 0 \end{array}\right], \mathbf{k}_{\text{cat}} = \begin{bmatrix} 10 \\ 100 \\ 8 \\ 4.55 \end{bmatrix}, \rho = 340$$

D

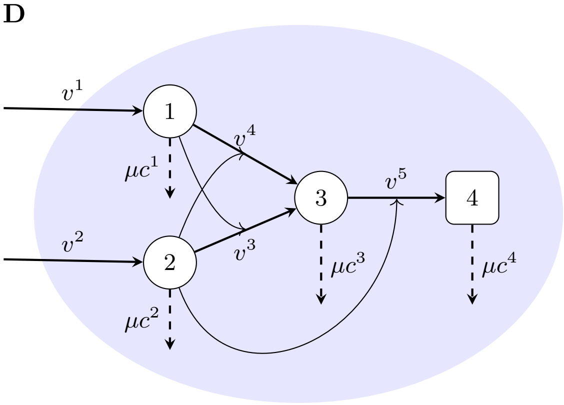

$$\mathbf{M} = \begin{bmatrix} 1 & 0 & -0.7 & -0.8 & 0 \\ 0 & 1 & -0.3 & -0.2 & -0.2 \\ 0 & 0 & 1 & 1 & -0.8 \\ 0 & 0 & 0 & 0 & 1 \end{bmatrix}, \mathbf{K} = \left[\begin{array}{ccccc} 1 & 0 & 0 & 0 & 0 \\ 0 & 1 & 0 & 0 & 0 \\ \hline 0 & 0 & 10 & 5 & 0 \\ 0 & 0 & 10 & 5 & 10 \\ 0 & 0 & 0 & 0 & 8.3 \\ 0 & 0 & 0 & 0 & 0 \end{array}\right], \mathbf{k}_{\text{cat}} = \begin{bmatrix} 15 \\ 10 \\ 10 \\ 9 \\ 4.55 \end{bmatrix},$$

$$\rho = 340$$

Fig. A. Schematics and parameters defining each model. For more details see the corresponding ods files in S1 File.

5. The dependence of λ on transporters

Equation 52 in the main text involves the sums $\gamma_j = \sum_i M^i_j$ of each column j in $\mathbf{M}$, which differ from zero only for transporters (Eq. 2 in the main text). This means Eq. 52 in the main text can be separated into distinct equations for $j = \mathrm{r}, e, s$

$$(\partial_{\mathrm{r}}\mu)\, f_{\mathrm{r}} = 0 \quad , \qquad [20]$$

$$(\partial_e\mu)\, f_e = 0 \quad , \qquad [21]$$

$$(\partial_s\mu)\, f_s + \lambda\gamma_s\, f_s = 0 \quad . \qquad [22]$$

From equations (20,21), we see that, at optimality, the summation in Eq. 53 in the main text can be simplified to a summation over s only

$$\lambda = -(\partial_s\mu)\, f^s \quad . \qquad [23]$$

Substituting now the partial derivative given by Eq. 27 in the main text, we obtain

$$\lambda = -\frac{\mu}{b^{\mathrm{a}}}\left(M^{\mathrm{a}}_s\, f^s - \mu\,\tau_s\, f^s - \mu\, f_l\, E^l_s\, f^s\right) \quad . \qquad [24]$$

The first summand in the parenthesis equals to zero, since transporters do not produce protein ($M^{\mathrm{a}}_s = 0$), and the second summand can be expressed in terms of protein fractions $\phi^s = p^s/c^{\mathrm{a}} = \mu\,\tau^s\, f^s/b^{\mathrm{a}}$, resulting in

$$\lambda = \mu\left(\sum_s \phi^s + \frac{\mu}{b^{\mathrm{a}}}\, f_l\, E^l_s\, f^s\right) \quad . \qquad [25]$$

6. Optimal enzyme concentrations and control coefficients

Equation 40 in the main text shares a striking analogy with an optimality condition for metabolic systems, established in (4): in an optimal metabolic state, maximizing a pathway flux at a limited total enzyme amount, the enzyme levels must be proportional to the flux control coefficients. This previous result reflects the assumption that the cell trades a cost (the sum of enzyme levels) against a benefit (the pathway flux), and that in an optimal state, the marginal cost and benefit, for any small change of enzyme levels, must be the same. Equation 40 in the main text, for optimal growth states, has a similar interpretation, but without an explicit benefit function for fluxes. Here, instead, if an enzyme level in reaction A has an indirect effect on a flux in reaction B and makes reaction B proceed more efficiently, then catalyzing enzyme for reaction B can be saved (and resources be redistributed to increase growth). Hence, we now have a trade-off between a cost (of investing enzyme in A) and an "opportunity benefit" (enzyme saved in reaction B). In the equation, an enzyme of interest (in reaction A) is described by p_e, its effect on all fluxes in the network (reactions B) is described by the control coefficients C^l_e, and the catalysts of these reactions are represented by p_l. By summing all marginal "opportunity benefits" $p_l C^l_e$, we obtain the equivalent to the marginal flux benefit in (4).

From the similar Eq. 34 in the main text, we can infer that at optimality, no two active enzymes can realistically catalyze the exact same reaction (i.e., have identical columns in $\mathbf{M}$). If this were the case, then both turnover times τ_e would have to be exactly the same, since the marginal opportunity would be identical for both enzymes (see Eq. 24 in the main text). This condition is highly unlikely to hold in realistic models, since real enzymes will always have different physical properties and therefore different kinetics. Thus, if several isoenzymes catalyze a particular reaction in a model, only one of these reactions will be active at optimality. The previous argument can be generalized to any two linear combinations of reactions in the model, and as a consequence, the submatrix resulting from restricting $\mathbf{M}$ to active reactions must have full column rank at optimal growth (see Refs. (2, 5, 6)). Note that in reality enzymes with very similar marginal values may still be expressed together due to the little selection pressure towards one of them. See model "D" on SI text "Examples of GBA models and R code for numerical optimization" for an example of a model with redundant reactions.

Table A. Amino acid frequency in the *E. coli* proteome at various growth conditions, calculated from protein sequence (retrieved from genome annotation of *E. coli* NC_000913.3 from RefSeq (7)) and weighted by protein abundance measured in Schmidt et al. (8).

Growth condition	A	C	D	E	F	G	H	I	K	L	M	N	P	Q	R	S	T	V	W	Y
Glucose	0.102	0.007	0.059	0.072	0.034	0.084	0.020	0.060	0.064	0.085	0.026	0.039	0.040	0.038	0.052	0.048	0.056	0.081	0.008	0.026
LB	0.102	0.007	0.058	0.074	0.033	0.084	0.019	0.060	0.068	0.081	0.026	0.038	0.039	0.036	0.055	0.046	0.056	0.083	0.007	0.025
Glycerol + AA	0.102	0.007	0.059	0.073	0.033	0.085	0.019	0.060	0.066	0.082	0.026	0.038	0.039	0.037	0.053	0.047	0.057	0.082	0.008	0.026
Acetate	0.105	0.009	0.059	0.069	0.035	0.084	0.019	0.059	0.061	0.085	0.025	0.039	0.041	0.041	0.047	0.049	0.057	0.078	0.009	0.028
Fumarate	0.104	0.008	0.059	0.070	0.034	0.084	0.019	0.059	0.063	0.085	0.026	0.039	0.040	0.039	0.049	0.049	0.056	0.079	0.009	0.027
Glucosamine	0.104	0.008	0.059	0.070	0.034	0.084	0.019	0.060	0.064	0.084	0.026	0.040	0.040	0.039	0.050	0.049	0.056	0.079	0.009	0.027
Glycerol	0.103	0.008	0.059	0.071	0.034	0.084	0.019	0.060	0.064	0.085	0.026	0.039	0.040	0.039	0.051	0.049	0.056	0.080	0.009	0.027
Pyruvate	0.103	0.008	0.059	0.071	0.034	0.085	0.019	0.060	0.063	0.086	0.026	0.039	0.041	0.038	0.049	0.049	0.056	0.080	0.008	0.027
Chemostat μ=0.5 h^{-1}	0.103	0.008	0.059	0.070	0.034	0.084	0.020	0.060	0.063	0.085	0.026	0.039	0.040	0.039	0.050	0.049	0.056	0.079	0.009	0.027
Chemostat μ=0.35 h^{-1}	0.105	0.008	0.059	0.069	0.035	0.084	0.019	0.059	0.062	0.085	0.026	0.039	0.041	0.040	0.048	0.049	0.056	0.078	0.010	0.028
Chemostat μ=0.20 h^{-1}	0.105	0.008	0.059	0.068	0.035	0.083	0.019	0.059	0.062	0.085	0.026	0.039	0.041	0.041	0.047	0.050	0.056	0.077	0.010	0.029
Chemostat μ=0.12 h^{-1}	0.106	0.008	0.059	0.068	0.035	0.083	0.019	0.058	0.061	0.085	0.026	0.039	0.041	0.041	0.047	0.050	0.057	0.077	0.010	0.029
Stationary phase 1 day	0.106	0.007	0.062	0.068	0.032	0.082	0.018	0.058	0.068	0.084	0.025	0.042	0.038	0.040	0.047	0.051	0.058	0.080	0.008	0.026
Stationary phase 3 days	0.105	0.007	0.062	0.068	0.032	0.083	0.018	0.059	0.067	0.084	0.025	0.041	0.038	0.040	0.047	0.050	0.058	0.080	0.008	0.026
Osmotic-stress glucose	0.103	0.008	0.060	0.071	0.033	0.083	0.019	0.059	0.065	0.084	0.025	0.040	0.040	0.038	0.050	0.049	0.057	0.080	0.009	0.027
42 °C glucose	0.102	0.007	0.060	0.073	0.033	0.084	0.019	0.060	0.065	0.084	0.026	0.039	0.039	0.038	0.052	0.048	0.057	0.080	0.008	0.026
pH6 glucose	0.103	0.008	0.060	0.070	0.033	0.083	0.019	0.059	0.065	0.084	0.025	0.040	0.039	0.038	0.050	0.049	0.057	0.080	0.009	0.026
Xylose	0.102	0.008	0.059	0.072	0.034	0.084	0.020	0.060	0.065	0.085	0.026	0.039	0.040	0.038	0.051	0.048	0.056	0.080	0.008	0.027
Mannose	0.104	0.008	0.059	0.071	0.034	0.084	0.019	0.059	0.064	0.085	0.026	0.039	0.040	0.039	0.049	0.049	0.056	0.079	0.009	0.027
Galactose	0.106	0.008	0.060	0.069	0.035	0.083	0.019	0.059	0.063	0.085	0.025	0.040	0.040	0.041	0.047	0.050	0.056	0.078	0.010	0.028
Succinate	0.104	0.008	0.059	0.070	0.034	0.084	0.019	0.060	0.064	0.085	0.026	0.039	0.040	0.039	0.050	0.049	0.056	0.079	0.009	0.027
Fructose	0.103	0.008	0.059	0.071	0.034	0.084	0.020	0.060	0.065	0.085	0.026	0.039	0.040	0.037	0.051	0.048	0.056	0.080	0.008	0.026
Coefficient of Variation:	1.25%	5.13%	1.48%	2.27%	2.44%	0.69%	2.61%	0.94%	2.87%	1.19%	1.01%	2.00%	1.82%	3.33%	4.15%	1.93%	1.09%	1.86%	7.55%	3.50%

References

1. W Liebermeister, E Klipp, Bringing metabolic networks to life: convenience rate law and thermodynamic constraints. *Theor. Biol. Med. Model.* **3**, 41 (2006).
2. H Dourado, MJ Lercher, An analytical theory of balanced cellular growth. *Nat. Commun.* **11**, 1226 (2020).
3. SB Zimmerman, SO Trach, Estimation of macromolecule concentrations and excluded volume effects for the cytoplasm of escherichia coli. *J Mol Biol* **222**, 599–620 (1991).
4. E Klipp, R Heinrich, Competition for enzymes in metabolic pathways:: Implications for optimal distributions of enzyme concentrations and for the distribution of flux control. *Biosystems* **54**, 1 – 14 (1999).
5. S Müller, G Regensburger, R Steuer, Enzyme allocation problems in kinetic metabolic networks: Optimal solutions are elementary flux modes. *J. Theor. Biol.* **347**, 182 – 190 (2014).
6. MT Wortel, H Peters, J Hulshof , B Teusink, FJ Bruggeman, Metabolic states with maximal specific rate carry flux through an elementary flux mode. *The FEBS J.* **281**, 1547–1555 (2014).
7. NA O'Leary, et al., Reference sequence (RefSeq) database at NCBI: current status, taxonomic expansion, and functional annotation. *Nucleic Acids Res.* **44**, D733–D745 (2015).
8. A Schmidt, et al., The quantitative and condition-dependent escherichia coli proteome. *Nat. Biotechnol.* **34**, 104 EP – (2015).
