# Supplementary figures and images for: Mathematical properties of optimal fluxes in cellular reaction networks at balanced growth
Source: PLoS Comput Biol. 2023 Jun 6;19(6):e1011156. doi: 10.1371/journal.pcbi.1011156 (PMC10275479; doi:10.1371/journal.pcbi.1011156)

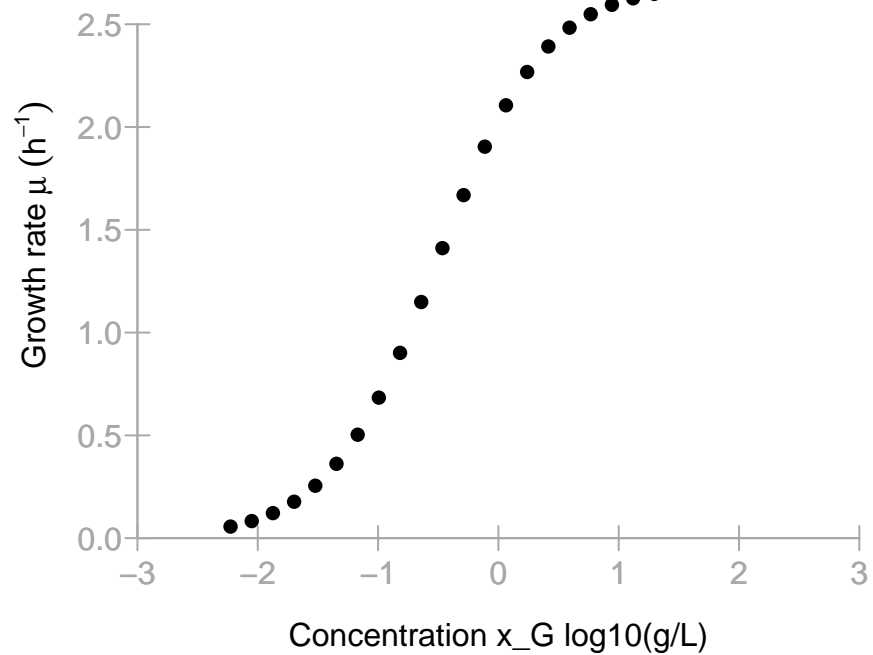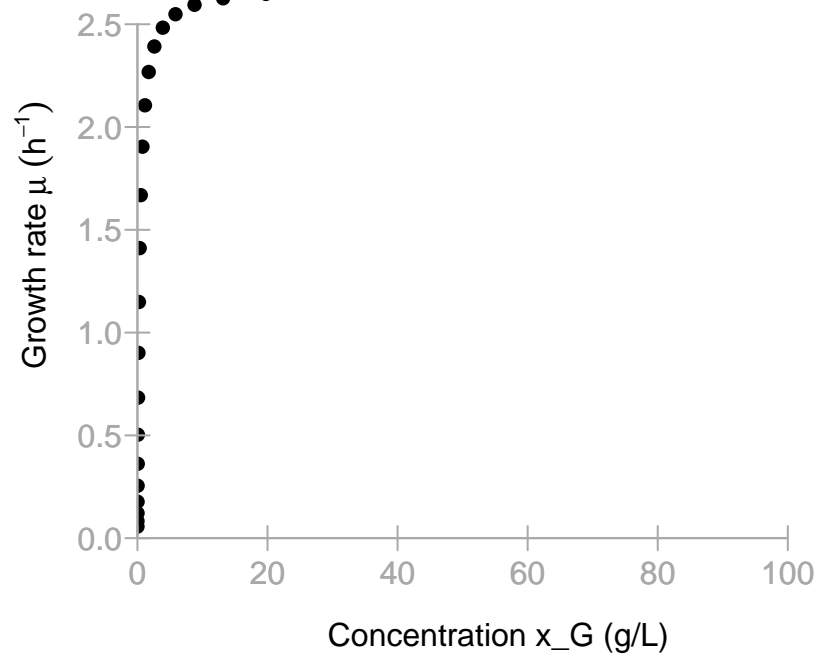

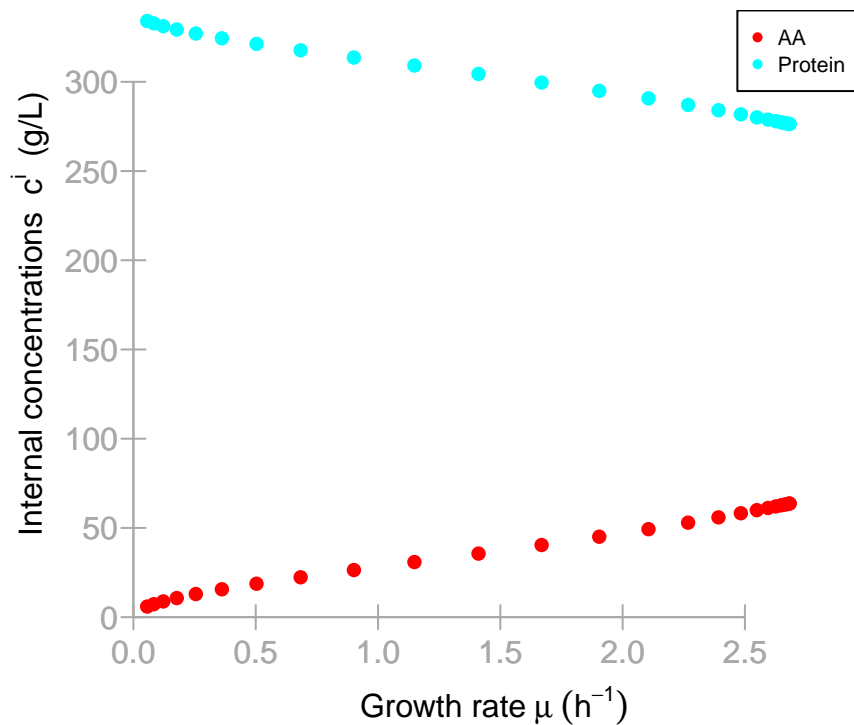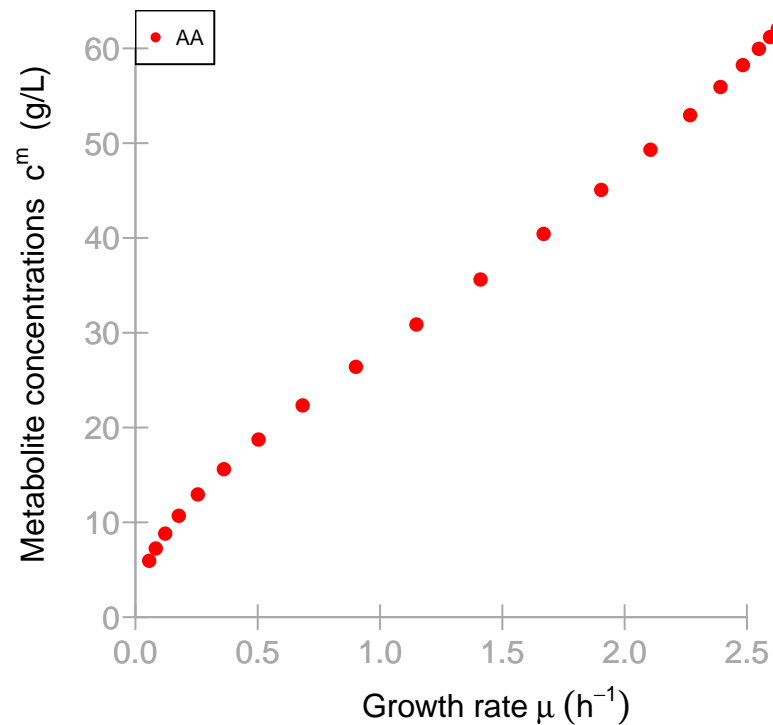

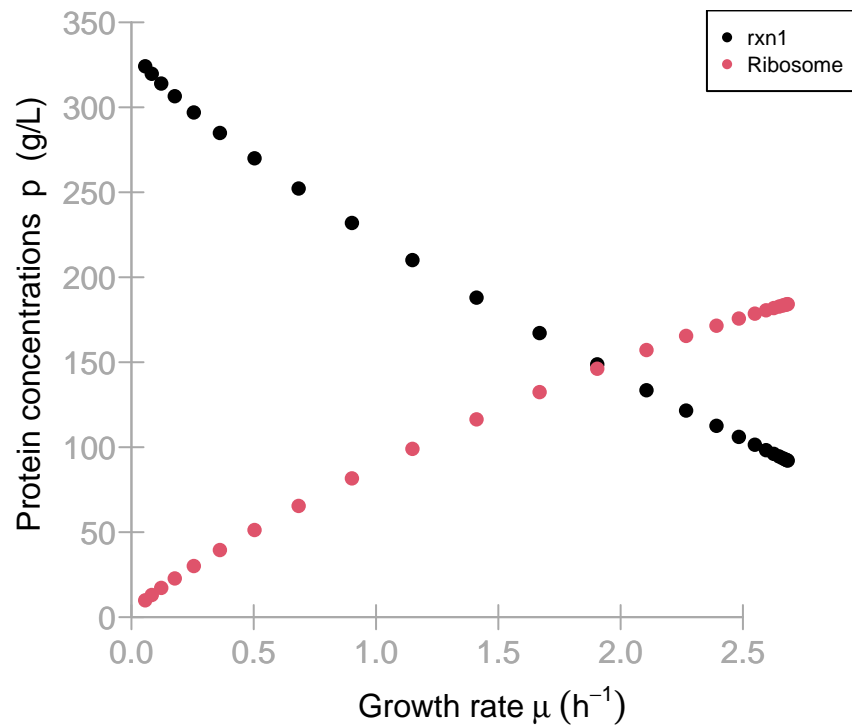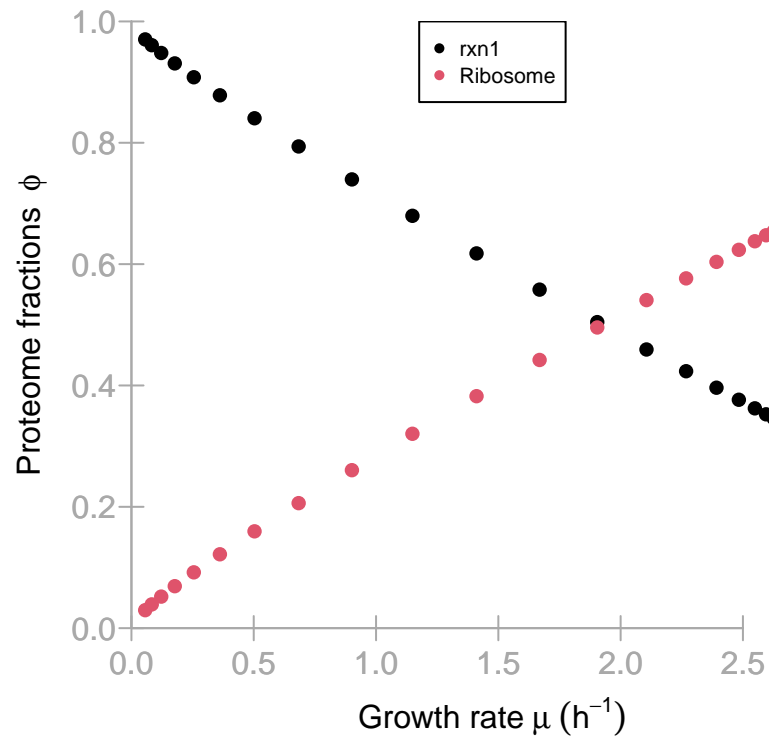

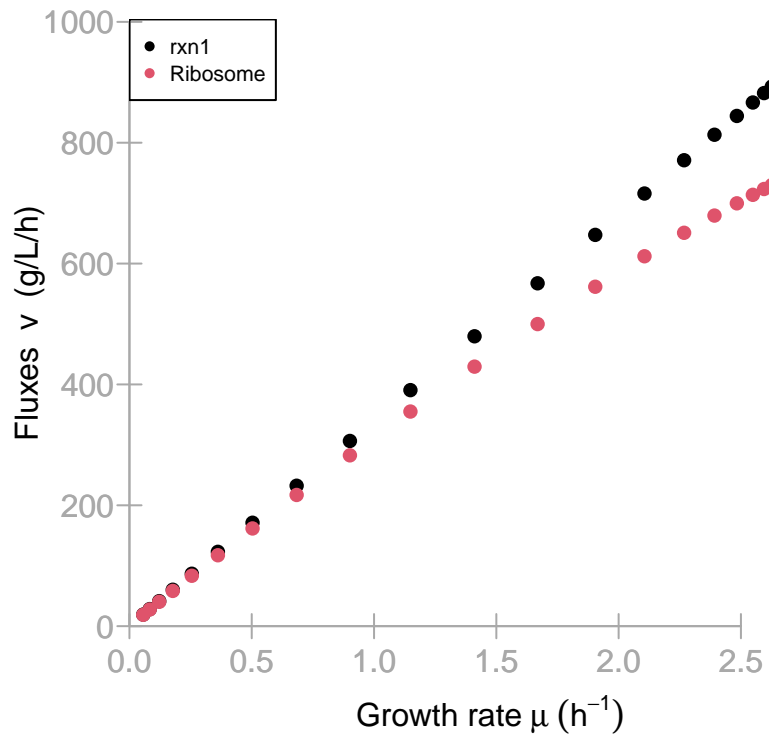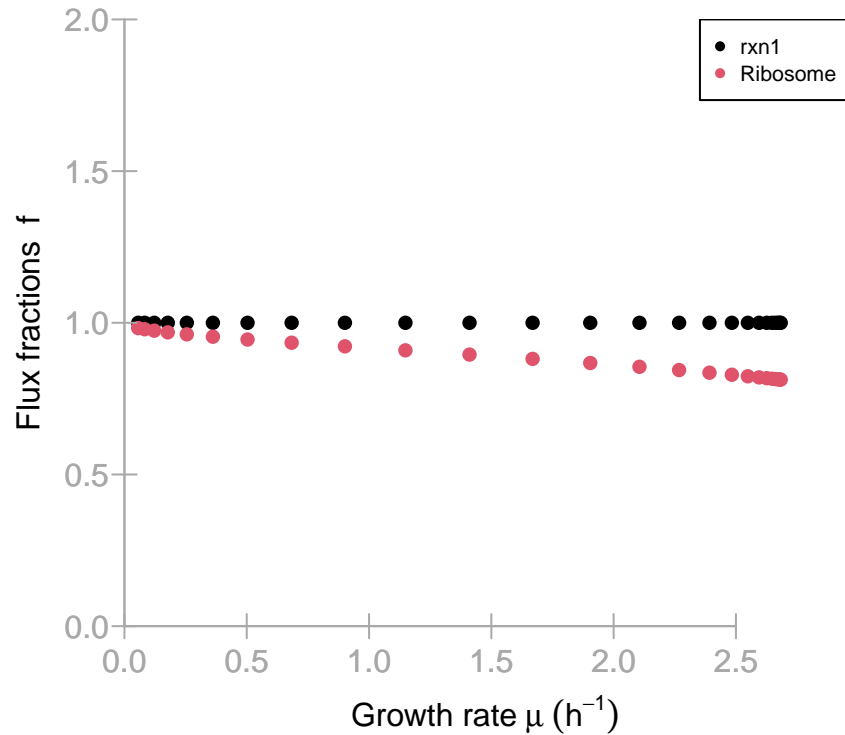

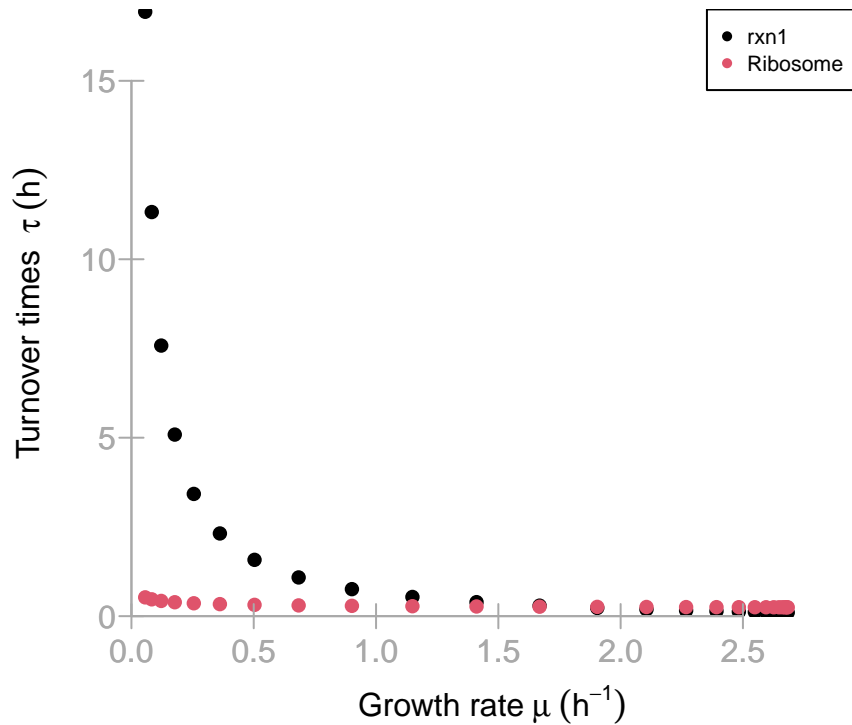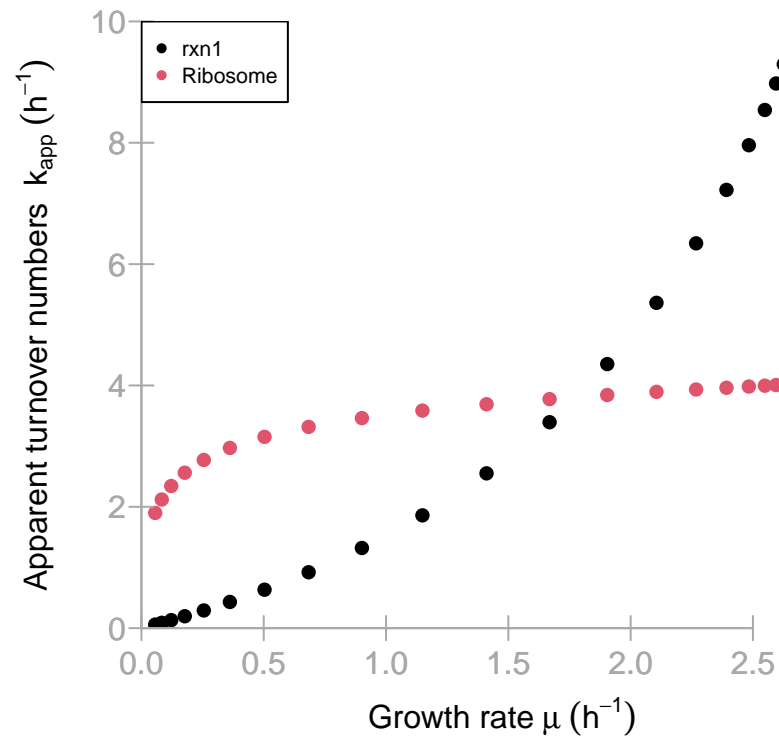

Supplement: S1 File — As described in the S1 Text. (ZIP) [file pcbi.1011156.s002.zip › Model A, mean time (0.0216s) results.pdf]

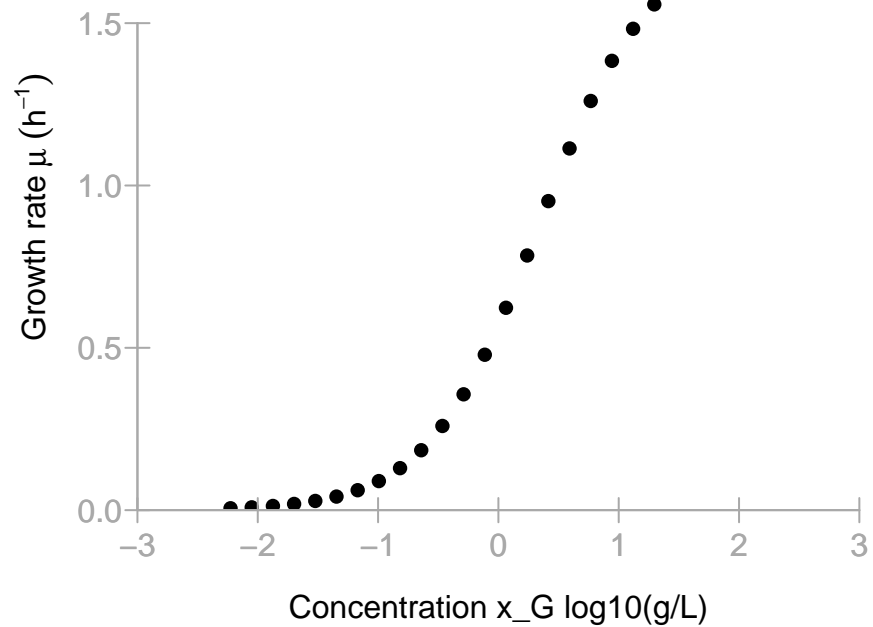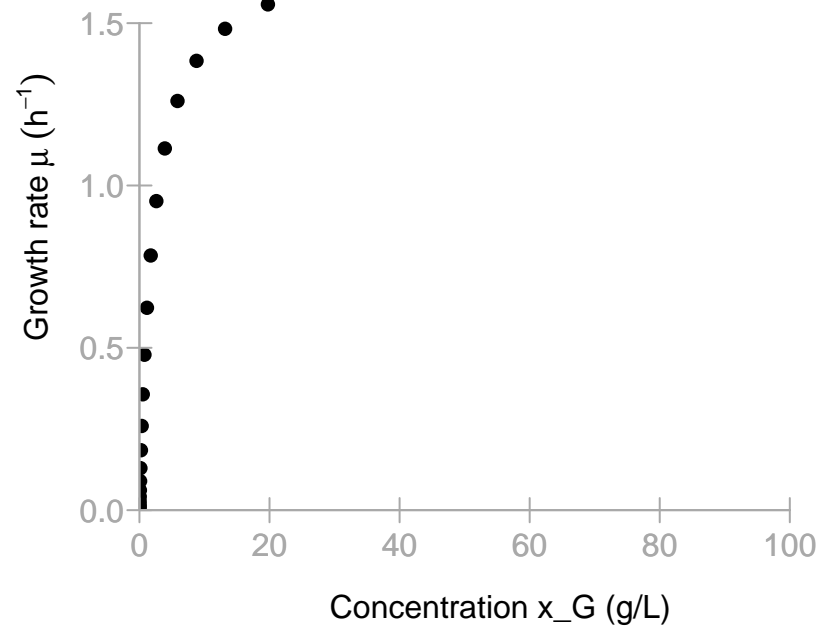

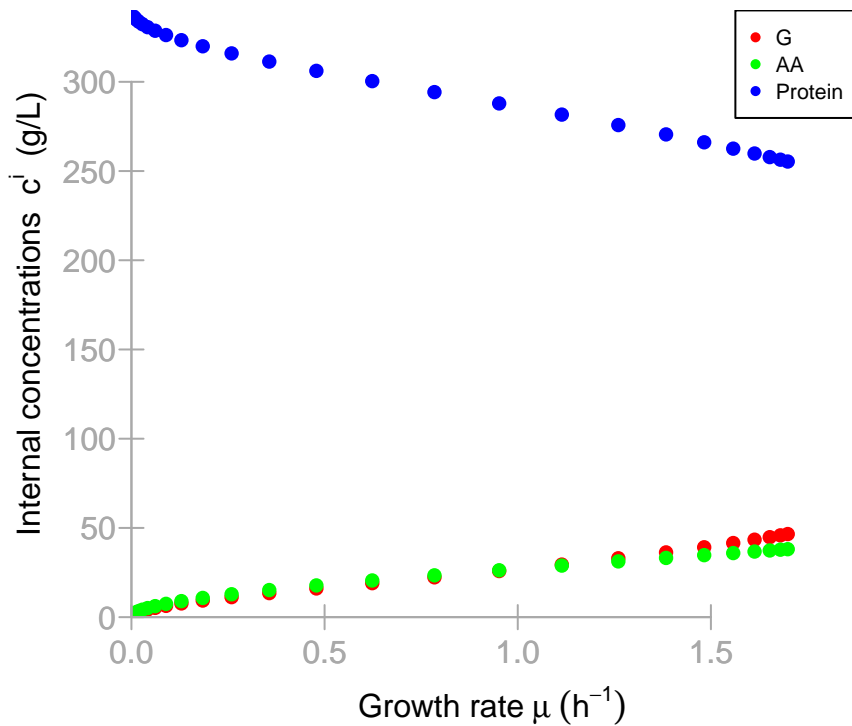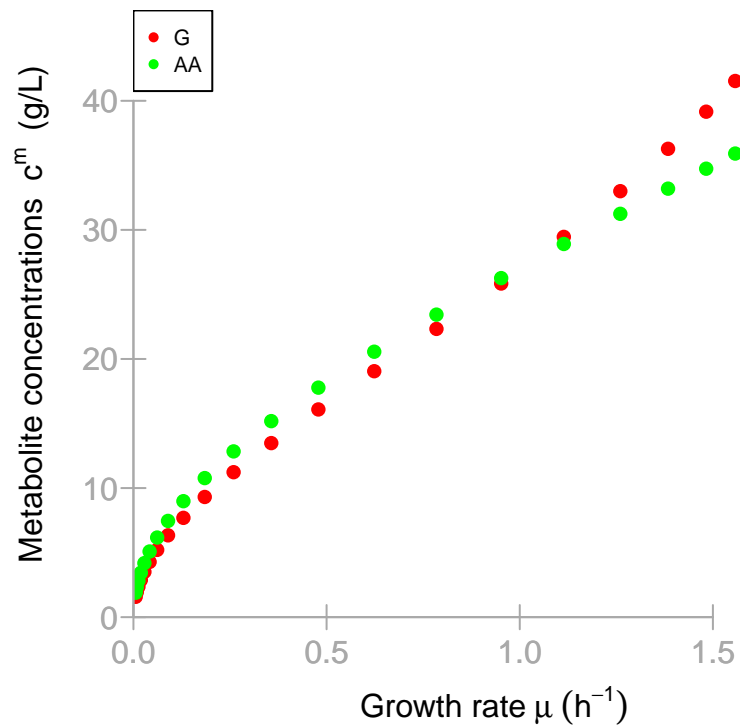

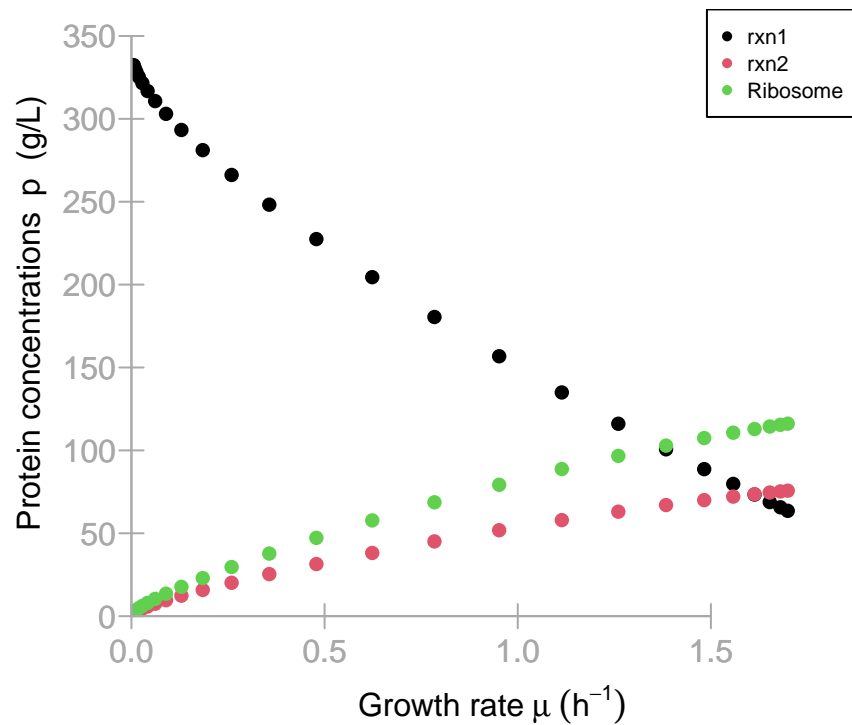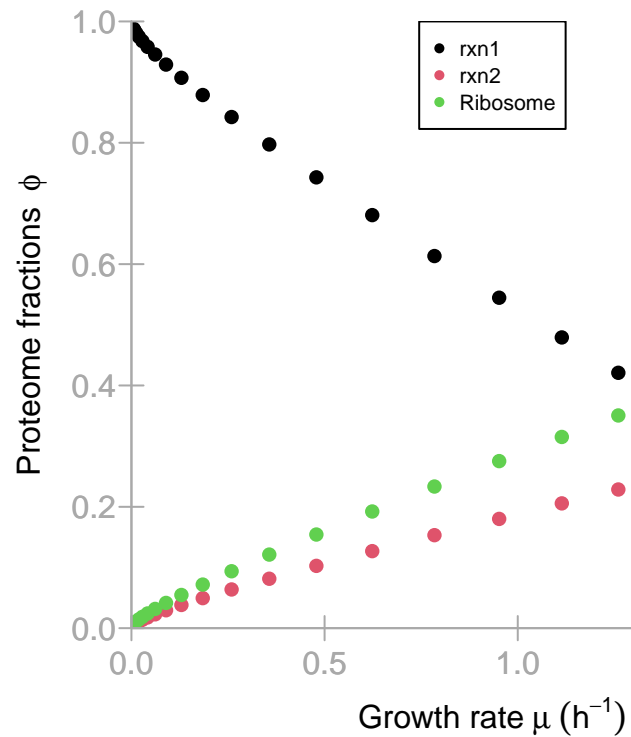

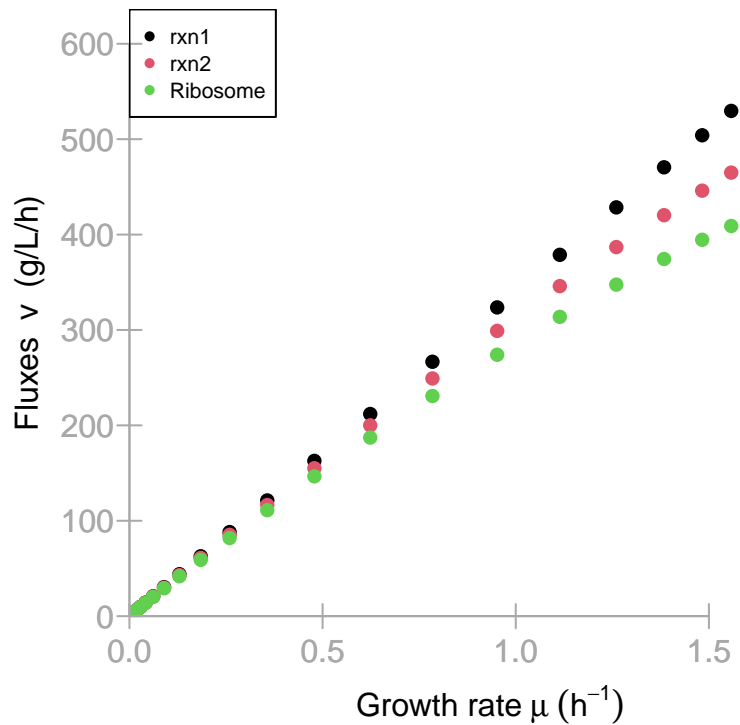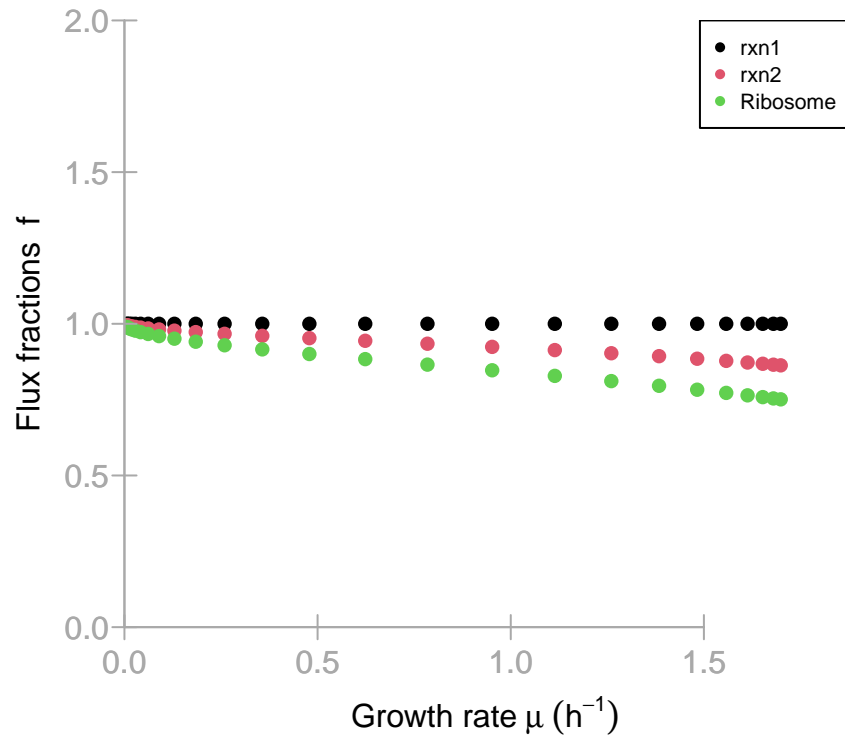

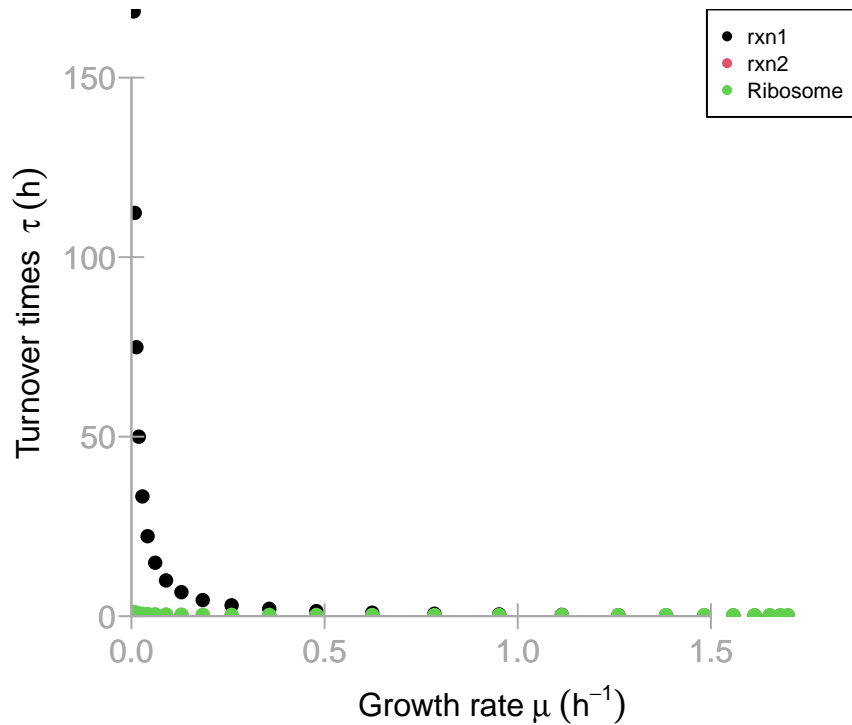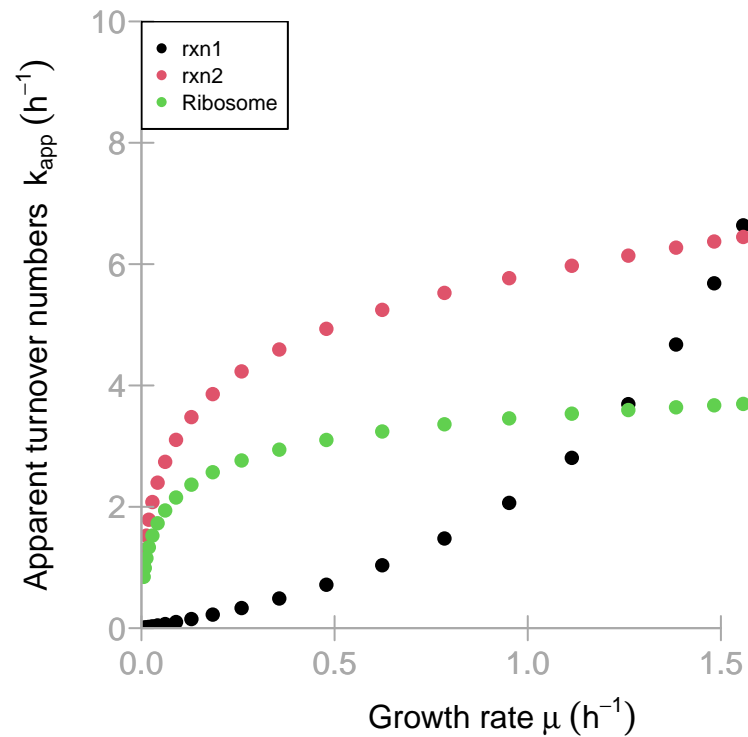

Supplement: S1 File — As described in the S1 Text. (ZIP) [file pcbi.1011156.s002.zip › Model B, mean time (0.0272s) results.pdf]

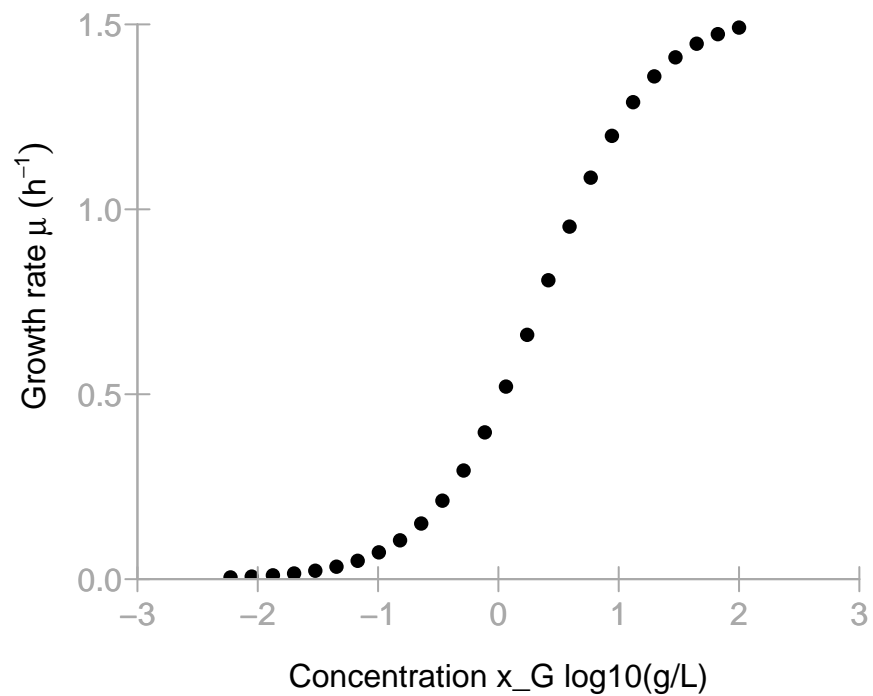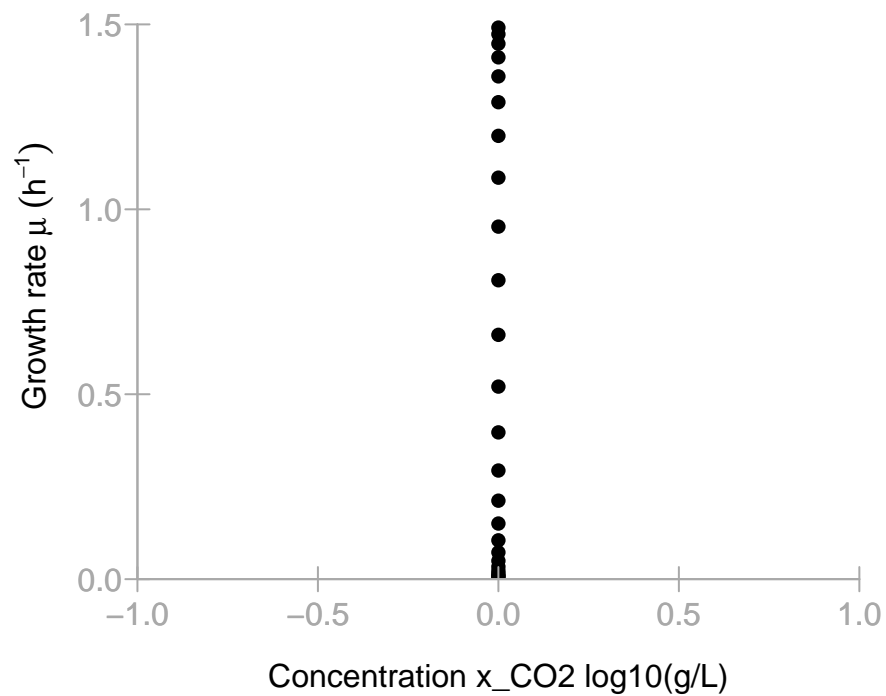

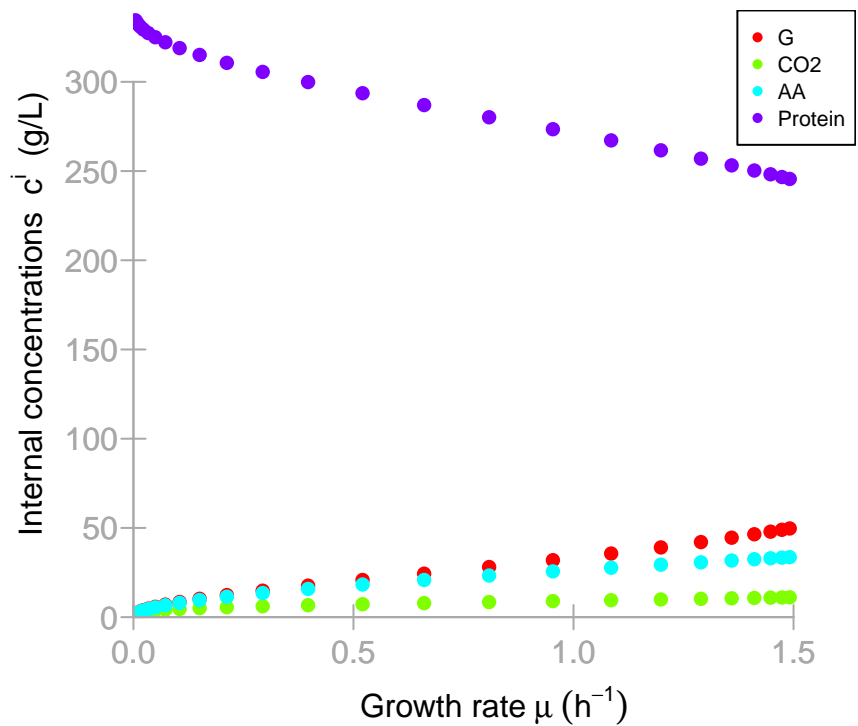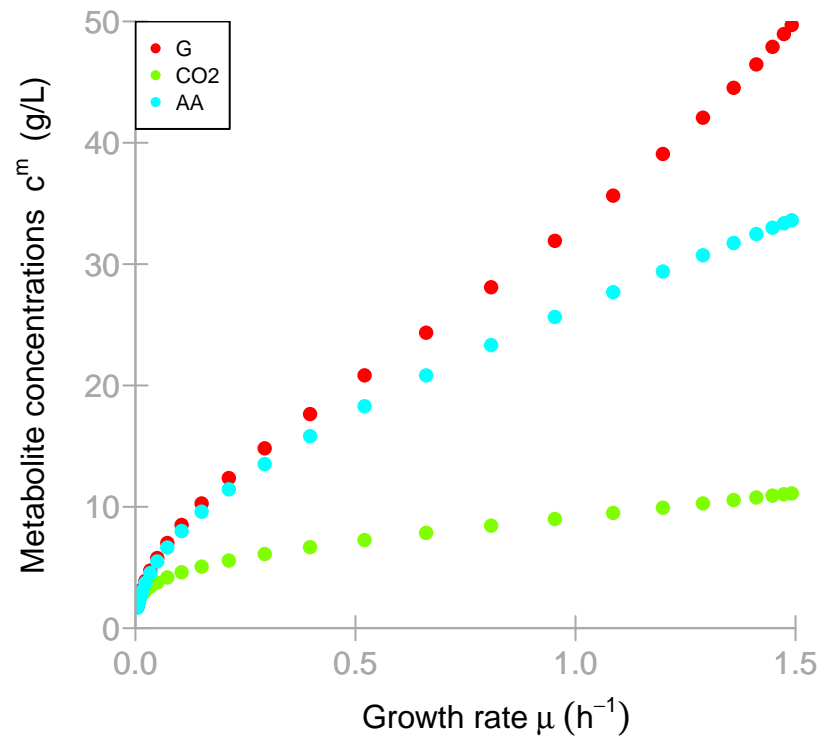

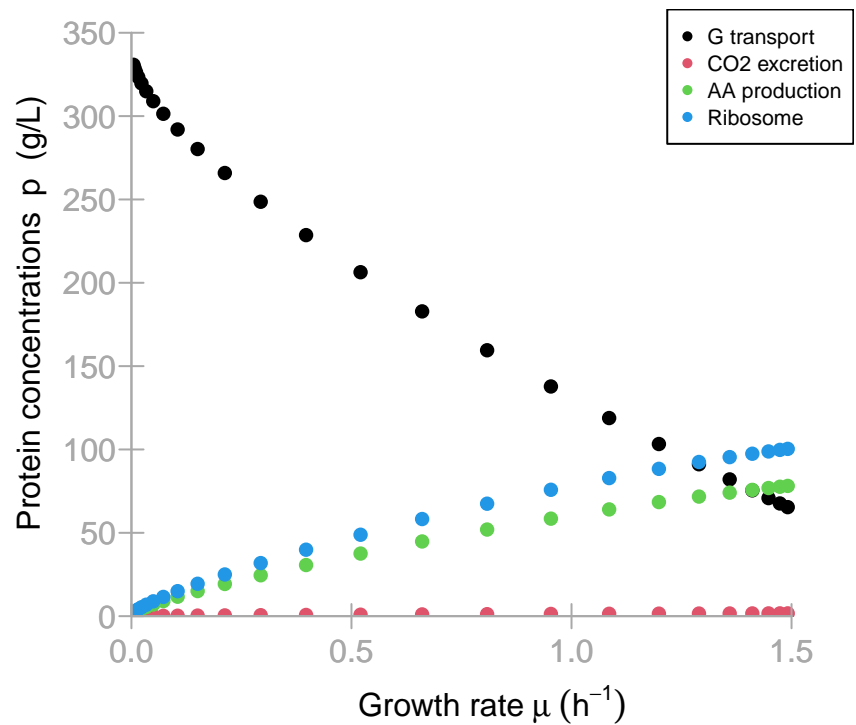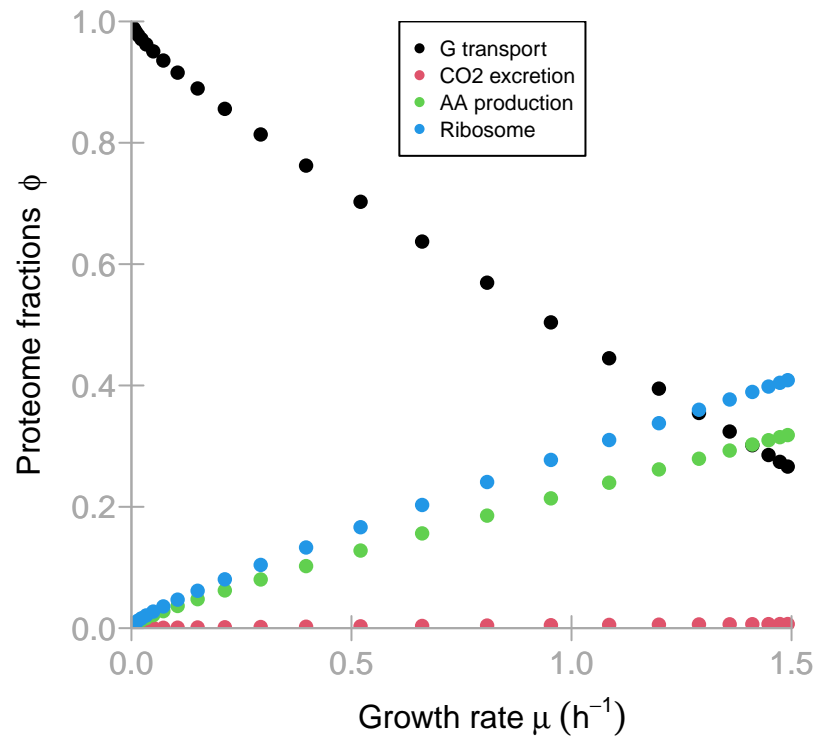

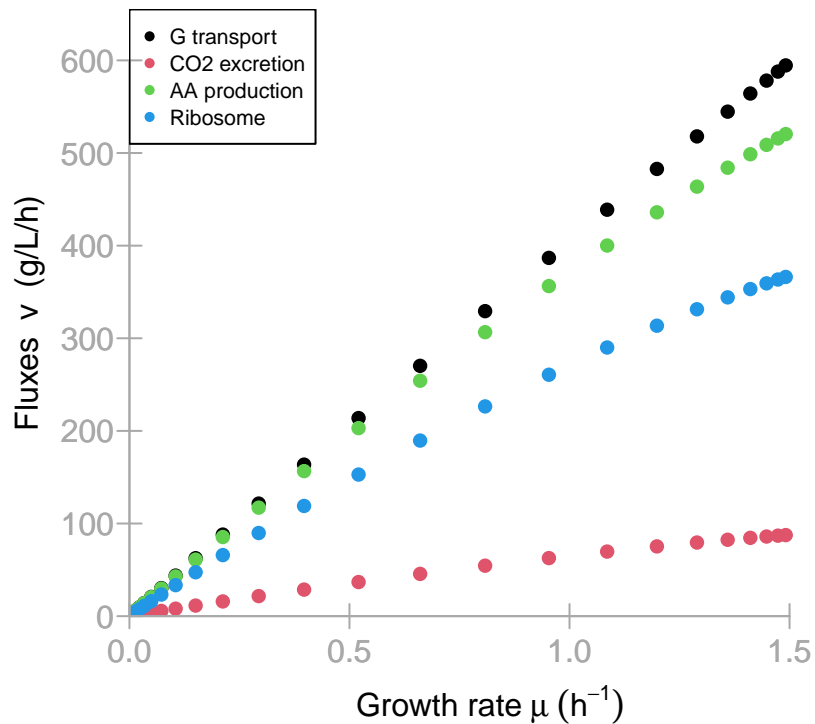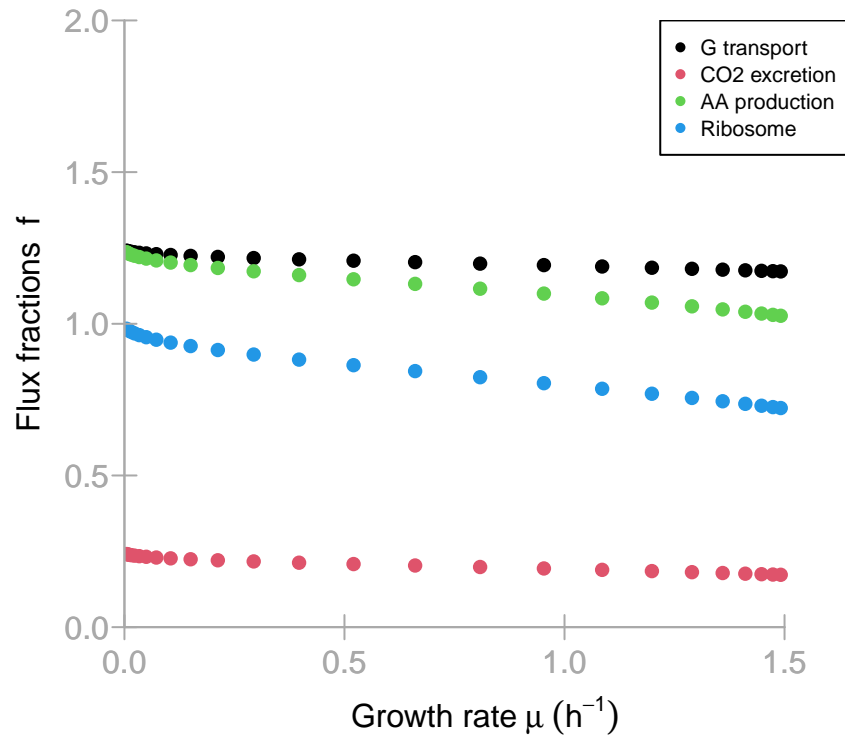

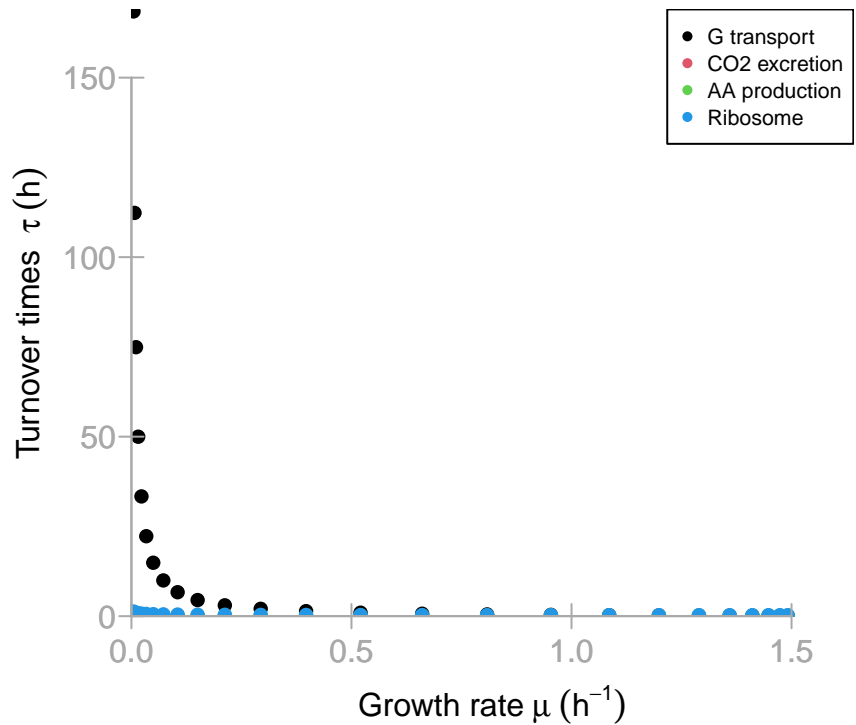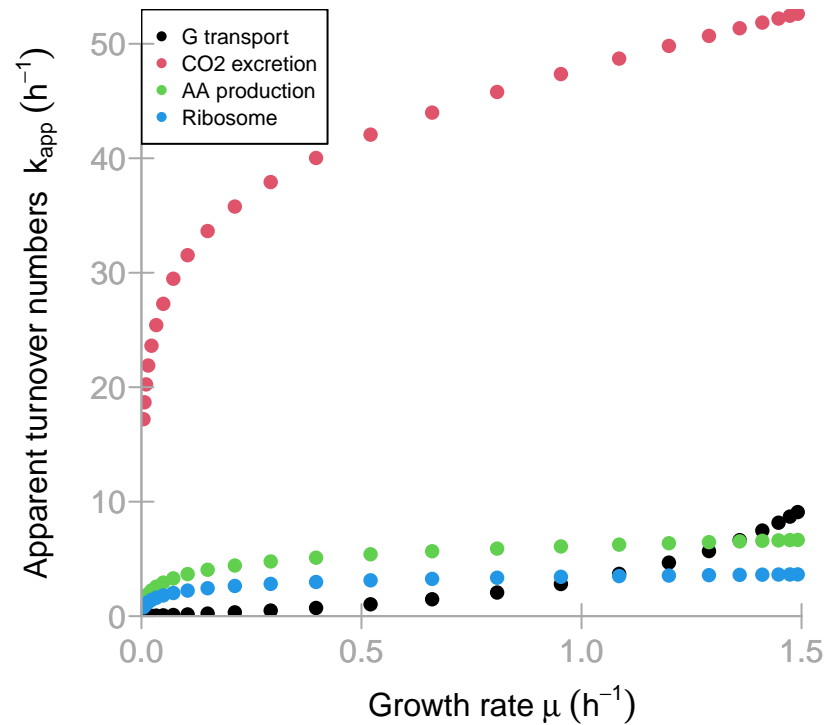

Supplement: S1 File — As described in the S1 Text. (ZIP) [file pcbi.1011156.s002.zip › Model C, mean time (0.0372s) results.pdf]

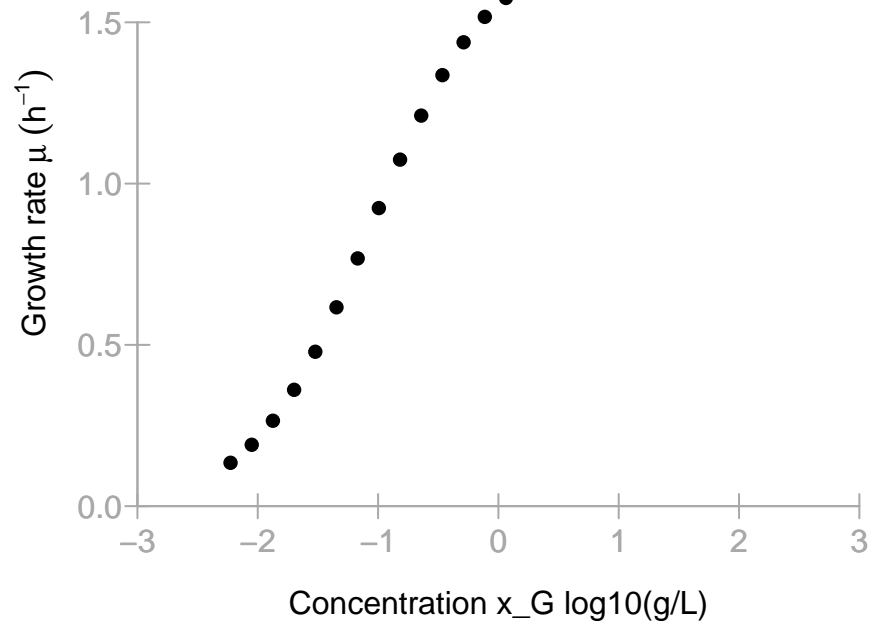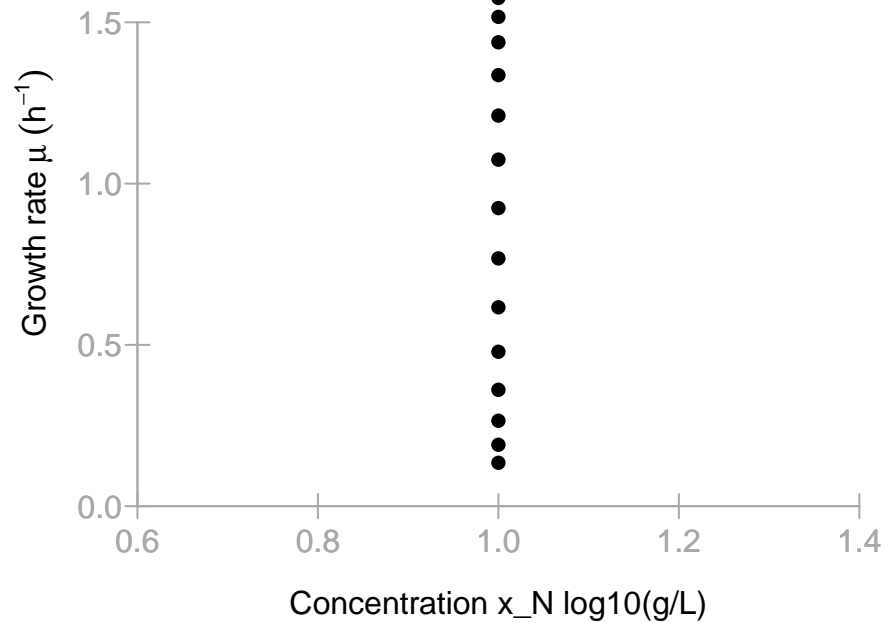

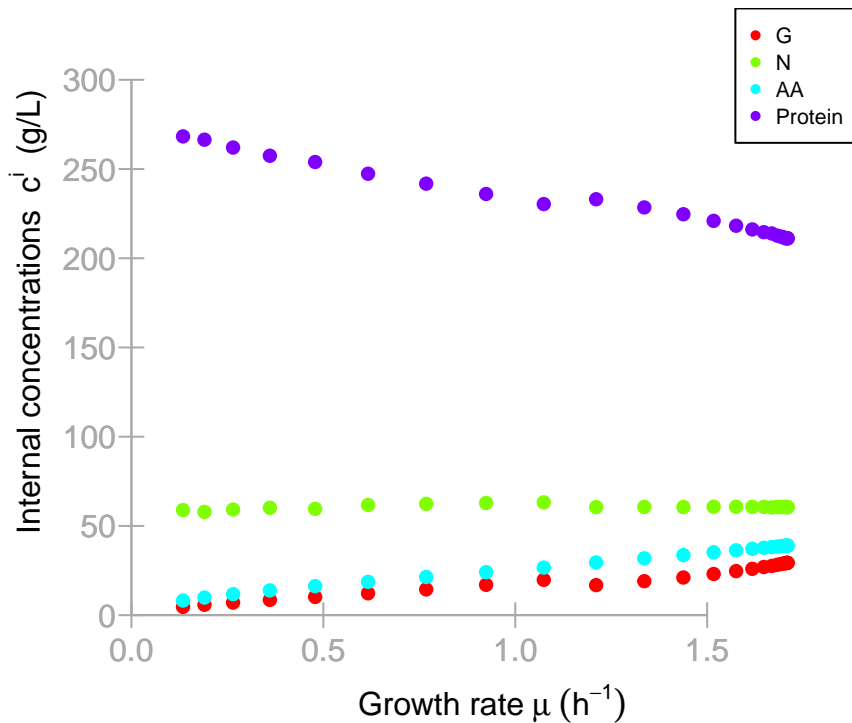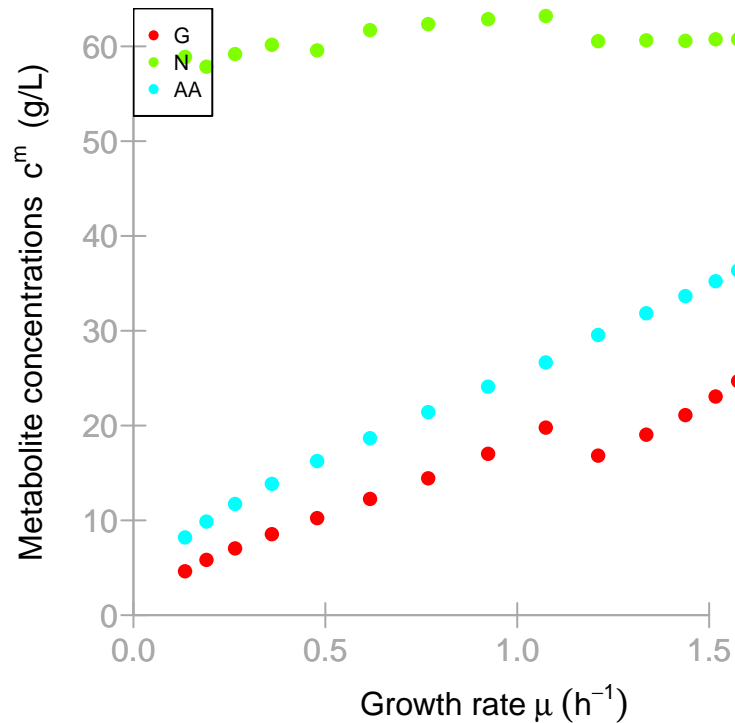

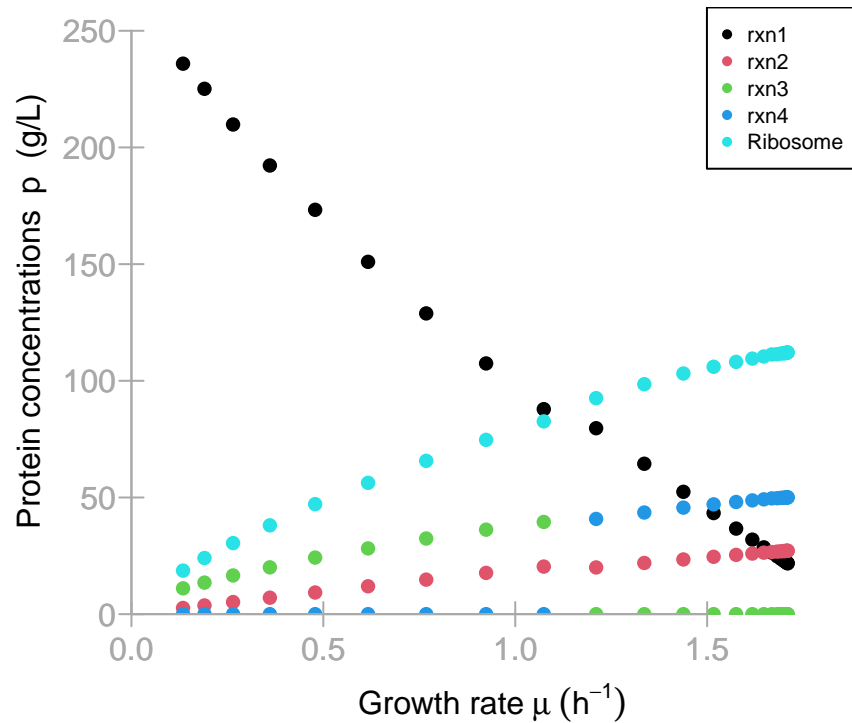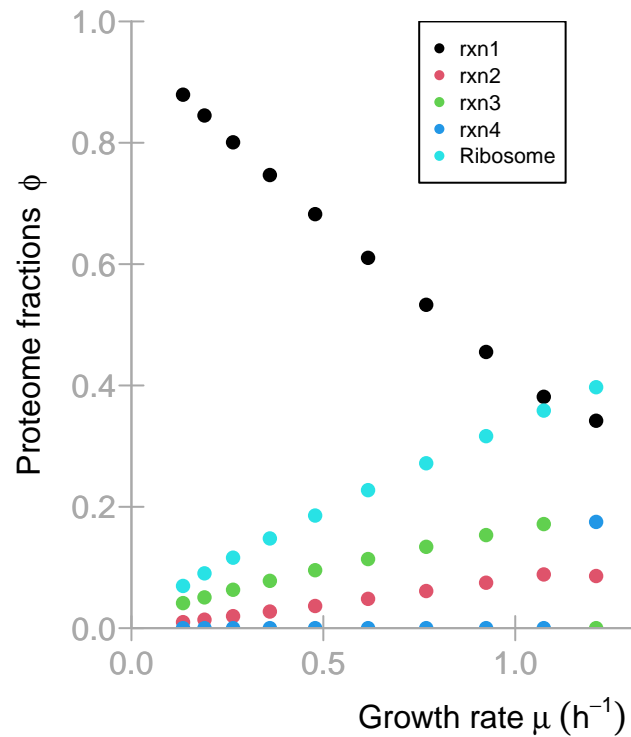

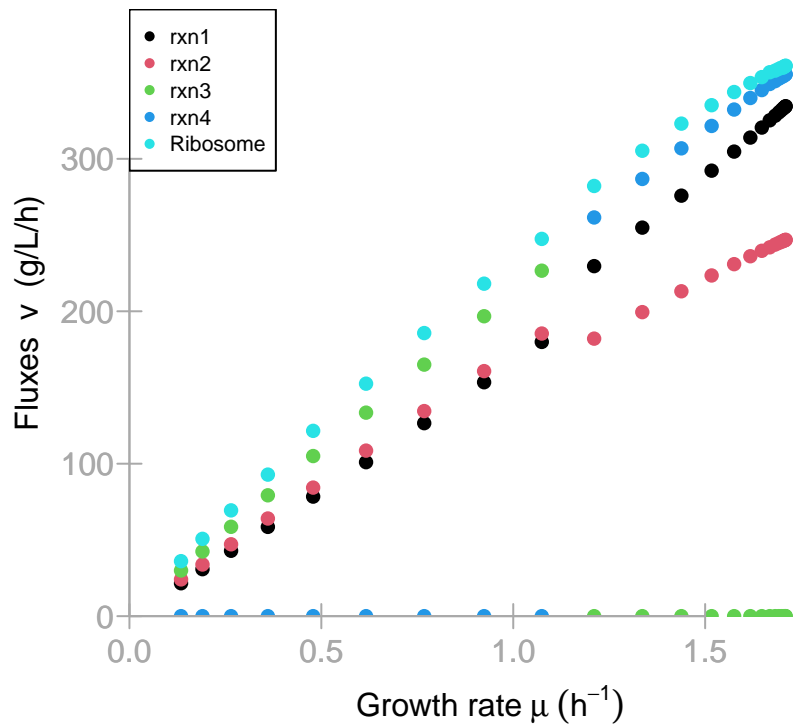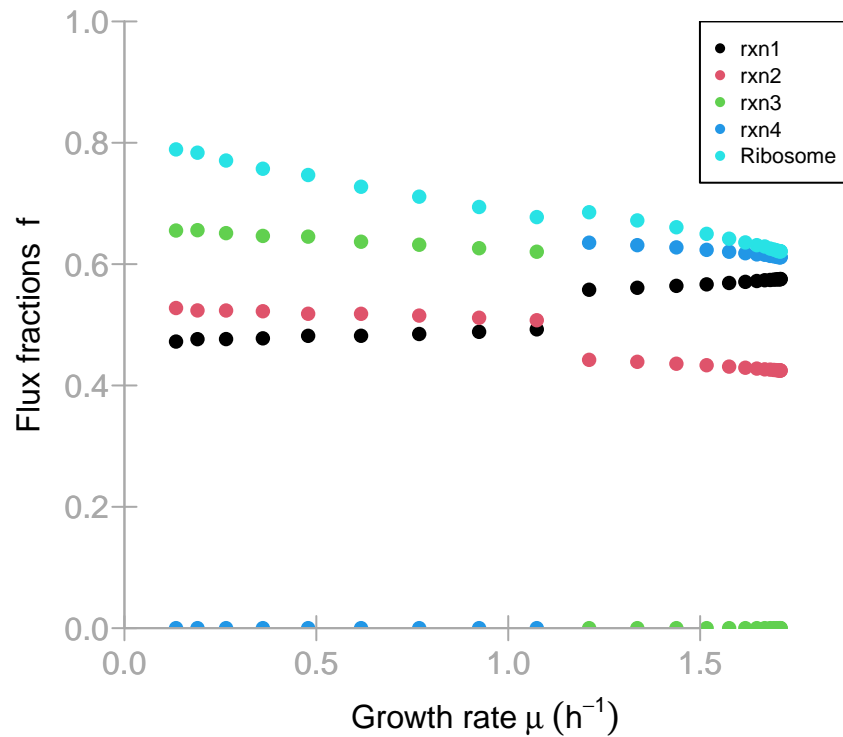

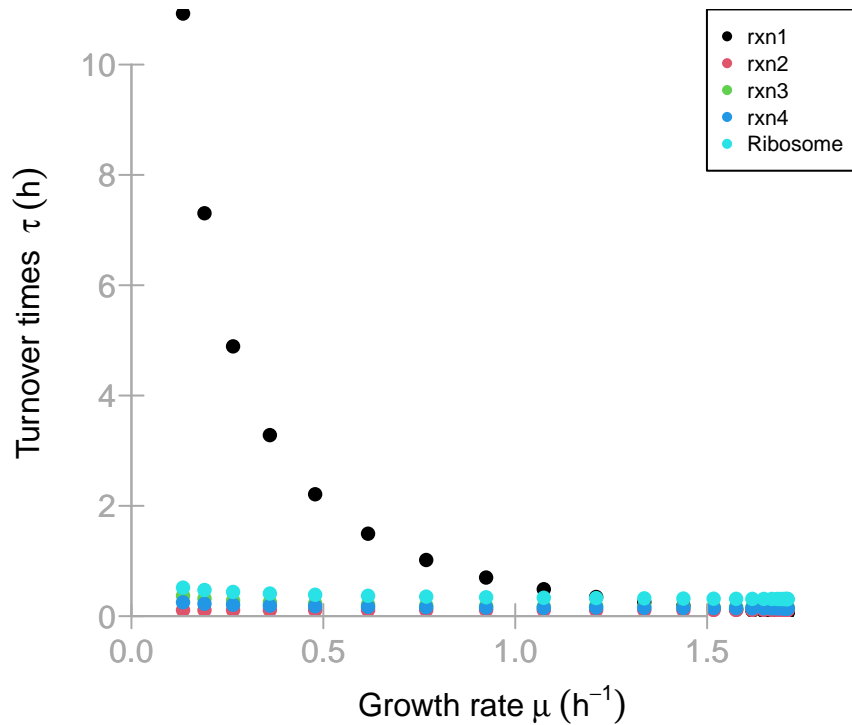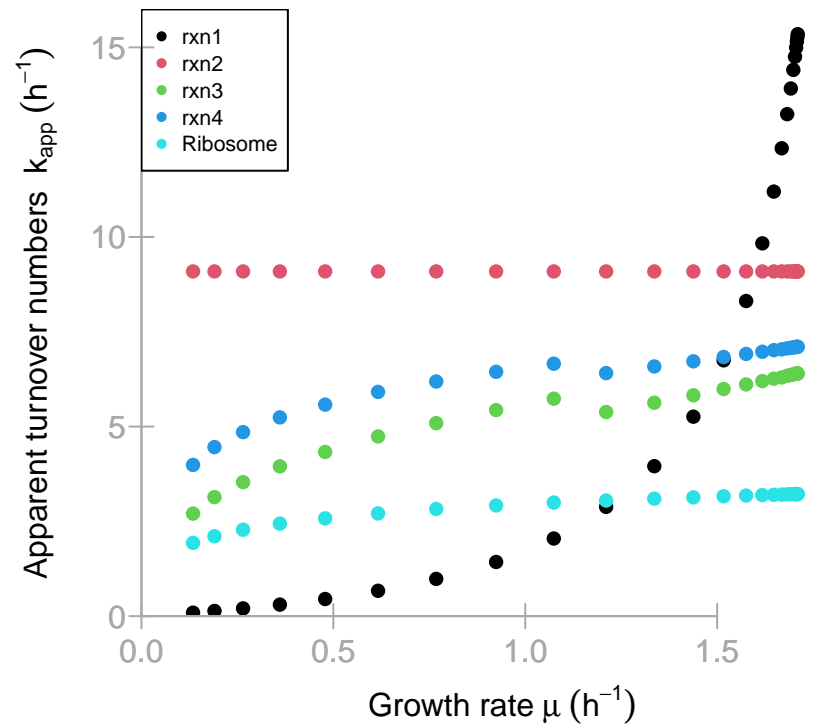

Supplement: S1 File — As described in the S1 Text. (ZIP) [file pcbi.1011156.s002.zip › Model D, mean time (0.08s) results.pdf]
