# Supplementary material for: Mathematical properties of optimal fluxes in cellular reaction networks at balanced growth
Source: PLoS Comput Biol. 2023 Jun 6;19(6):e1011156. doi: 10.1371/journal.pcbi.1011156 (PMC10275479; doi:10.1371/journal.pcbi.1011156)
Supplement: Attachment — Submitted filename: Response to Reviewers.pdf [file pcbi.1011156.s003.pdf]

Response to Reviewers

Reviewer #1

Summary:

In this work, the authors introduce a mathematical method to perform growth balance analysis based on a known method to solve convex nonlinear optimization. The current manuscript only focuses on the mathematical formulation but does not provide biologically relevant examples to demonstrate the usefulness of the formulation. The manuscript often reads as lacking in essential detail. Comments are also provided below after the reviewing process. In addition, the reviewer suggests that the authors move detailed mathematical derivations and formulation to Supplementary Methods and focus on explaining the formulation (i.e., so as to help readers understand rather than derive the formulation).

Response: We appreciate the reviewer's input, and made many improvements to the manuscript based on it (see details below). We agree that some detailed mathematical derivations are better moved to the supplement. Moreover, the importance of the present work indeed needs to be communicated better to an audience beyond a small number of specialists. As suggested by the Reviewer, this communication would be aided through the careful construction and analysis of a biologically relevant example – which would involve the choice of reactions necessary to model a realistic growing cell, the appropriate nonlinear kinetic functions of these reactions, and the corresponding kinetic parameters. However, constructing such an example constitutes a substantial scientific project in itself. In contrast, the present work is designed as a theoretical study that focuses entirely on a mathematical effort: (i) to unify previous theoretical frameworks to model and analyze metabolism into a single, fundamental theory; (ii) to then extend those frameworks to even more elaborate, realistic models that account for the dilution by grow of all cellular components and thus capture all important trade-offs related to the cell resource allocation; (iii) to derive, for the first time, the exact analytical solution to the general underlying mathematical problem describing growing cells; and finally (iv) to show how these derived mathematical properties of optimally growing cells relate to fundamental concepts of Metabolic Control Analysis and Metabolic Economics, extending also these important theoretical fields of study from non-growing steady states to the scenario of growing cells. All the mathematical derivations are built on only a few, basic assumptions: mass conservation, kinetic rate laws, and limited cell density. Importantly, these assumptions are shared by most commonly used metabolic and kinetic modeling frameworks, including all constraint-based methods that account for resource allocation. Our exact theoretical results are as relevant as their underlying assumptions, which are considered fundamental to model metabolism by a wide community of computational systems biologists. Thus, we strongly believe that our theoretical results are of intrinsic value to the wider community; future applications to realistic biological systems are a topic for separate follow-up studies. We regret that we have not made the purely theoretical purpose and merits of our study more explicit and accessible in the original submission.

Action: We moved the detailed mathematical derivation of Eqs.(26)-(30) and Eqs.(33)-(35) to the Methods section. We now make the purely theoretical purpose of our study explicit throughout the text; this clarification includes changing the title to "Mathematical properties of optimal fluxes in cellular reaction networks at balanced growth". We carefully checked that all essential details are included in the main text (see also our replies to specific comments below).

Major concerns

1) The introduction appears to present only a partial picture of the proposed GM framework with respect to other, non-linear, metabolic modeling frameworks, where many are concerned with the paucity of information needed to construct a kinetic model. Frameworks such as Ensemble Models (EMs), GRASP, iSCHRUNK (within ORACLE), and K-FIT are used to overcome this barrier. Is this not also a barrier to the GBA/GM frameworks? Other common terms in linear modeling frameworks, such as genome-scale models (used in the discussion, line 590) should be introduced here if used elsewhere.

Response: While the paucity of kinetic parameters is indeed an important problem in the construction of kinetic models, it is not the problem we are addressing in the present study. The estimation and/or fitting of kinetic parameters represents a parallel problem that may be addressed by other methods, including Machine Learning, as we indicate in our discussion (line 648). We introduce the term "genome-scale model" on line 43.

Action: None.

2) Do elements of vector v in M*v = mu*c (Eq. 3) correspond to fluxes of the reactions facilitated/catalyzed by transporters (transport reaction), enzymes (metabolic reaction), and ribosomes (protein translation reaction)? Or do those elements in v correspond to those machineries' synthesis fluxes? If the former is true, then how are the machiney's synthesis fluxes accounted for in the formulation?

Response: The first: the elements in **v** are reaction fluxes facilitated by transporters, enzymes, and ribosomes. The fluxes **v** have the same usual meaning as in for example FBA. The machinery is assumed to be made entirely of protein (a restriction that can be generalized, see line 156). The corresponding synthesis fluxes are accounted for through the total production of protein by the ribosome, which offsets the dilution of total protein by growth, $v^r = \mu c^a$, just as the synthesis fluxes of reactants offset their dilution by growth.

Action: We now more clearly state these relationships on line 217.

3) In addition, it is unclear to the reviewer on what constraints are formed from the column "r" and row "a" of M. On protein and ribosome, total protein concentration is constrained but to be less than a variable "c"? Then, "c^a" is constrained to be less than "rho". Is "rho" a constant/parameter? What about ribosome concentration? Does this mean the model's

maximal growth rate is limited by other constraint(s) rather than protein and ribosome availability (as proteins and ribosomes can be produced as much as the cells need)?

Response: rho is a constant parameter, as stated on line 183. Per definition, c^a is the total protein concentration (see equation (5))**.** The model's maximal growth rate is constrained mathematically by the interplay of *all* constraints in equations (3)-(8). The explicit relationship between the maximal growth rate and these constraints is the main focus of our paper, where we disentangle this relationship by reformulating the problem only in terms of flux fractions **f,** which in turn uniquely determine all concentrations and fluxes. We do not constrain the total protein concentration (c^a) to a fixed value, but only that the sum of proteins (defined by equation 5) is connected to the mass conservation via the corresponding row "a" in equation (3) and connected to the total density via equation (6).

Action: We clarified these issues by adding corresponding explanations on lines 131 and 217.

4) Line 183-184, the statement "The property (2) guarantees mass conservation within reactions..." is not true as M is derived from S. The two sets of positive and negative coefficients in M were mentioned to be normalized on individual reactions. In other words, the two sums of molecular weight of positive and negative terms, respectively, can differ (which indicate reaction is mass, and elemental, imbalances).

Response: It is true that in most metabolic models, the stoichiometric matrix **S** does not fully conserve mass, as it often ignores some common reactants such as water, and as it includes exchange reactions. However, we demand that **M** accounts for all reactants and initially considers the full, closed system. Thus, **M** guarantees mass conservation within reactions by construction.

Action: we added a sentence on line 197 to clarify this.

5) To create Eq. 11, does this mean that the growth rate "mu" has to be constant? Thus, to solve a GBA optimization (previous or current formulation), does "mu" need to be set to a constant? If so, how could one solve for the maximal "mu" in GBA?

Response: Eq. 11 holds for any μ, so μ does not have to be set to a constant. The current paper treats a cellular system in quasi-steady state, i.e., we assume that the growth rate μ is constant in time. However, μ is a free variable of our mathematical formulation, and is not set to a constant. Instead, we derive here (for the first time) the growth function (16) that determines the explicit dependence of μ on the cellular state **f**, facilitating the maximization of μ.

Action: None.

6) Line 222, the authors state that extracellular concentrations, "x", are constant. Is this assumption compatible with simulation for batch conditions (i.e., nutrients are depleted over time)?

Response: The assumption that **x** is constant in time is compatible with the balanced growth (or quasi steady-state) assumption, which is the topic of our manuscript and which is common to all

widely adopted frameworks, such as FBA or RBA. For batch conditions, the balanced growth assumption is clearly not valid in general, since changes in environmental conditions inevitably cause changes in the cellular state and in the (specific) growth rate. The possible extension to a dynamical theory of growth in changing environments is discussed on line 737.

Action: We now introduce **x** on line 125 by stating that they are "fixed and given".

7) Based on Eq. 19, are flux fraction "f_j" variables positive? What about reversible reactions? Also, could the Eq. 19 constraint be replaced by the "f_j >= 0" constraint because "tau_j" are positive? If not, could the authors provide a comment on the current form of Eq. 19?

Response: We agree with the Reviewer that we should have explained the sign of "f_j" more clearly. We assume that the flux fractions"f_j" may have any real value, including negative when the reaction proceeds backwards. The "tau_j" can also take any real value, except from zero. The non-negativity constraint on protein concentrations (equation (8)) means that "f_j" and "tau_j" must have the same sign (equation (21)).

Action: We clarify the sign of **f** on line 249, and add a discussion on the reversibility of reactions on line 326.

8) It would be helpful to readers to include in the growth problem (Eq. 21) notation to indicate the variables' domains (e.g., are flux variables strictly non-negative?). This could also be helpful in understanding how to construct models which can use this framework (e.g. are reversible reactions allowed?).

Response: We agree with this suggestion. The only free variable in equation (21) is **f**, and its domain is R^N.

Action: We added the domain on equation (21), and now define N as the number of reactions on line 323.

9) The reviewer does not find support for the statement presented in lines 605 to 607: "These conditions are local for each reaction, i.e., they do not require complete knowledge of the cellular reaction network and its kinetics." Without complete knowledge, how are M and "tau" defined? In this work, overall, there is no concrete, concise, statement or statements as to what knowledge is needed to build a model to which this analysis can be applied.

Response: Our use of the term "local" in line 606 is indeed confusing, because it does not mean the same here as it does when it is first used in line 476 (when referring to how equation (44) depends only on the local knowledge of the reaction network). We now restrict the use of "local" to the first case only. We do provide a concrete and concise statement as to what knowledge is needed to build a model in line 122. In line 331, we provide further discussion on the information necessary for determining the functions \tau.

Action: We updated line 660 to clarify that.

10) According to the abstract and title, the purpose of this manuscript is to introduce the Growth Mechanics (GM) framework, but the phrase "Growth Mechanics" is only used in the title and abstract, and the acronym is only used in the abstract and last paragraph of the discussion. What is the GM framework? How is it different from the GBA framework, and where are the generalizations which are mentioned in the abstract? How is it more powerful? Some discussion on the distinctiveness of the two frameworks and a comparison of their applications would be useful.

Response: We have to agree with the Reviewer that the distinction between the GBA and Growth Mechanics (GM) frameworks is confusing. We thus removed the term "Growth Mechanics" from the manuscript, and now only emphasize that the present study is an extension of the previous GBA framework

Action: We updated the title to more clearly convey the goal of this study, and excluded all references to "Growth Mechanics".

11) Marked inconsistencies exist in how "tau" is discussed. In Table 1, it is noted as a turnover time for the reaction, and later on discussed as the inverse of the usual factor in kinetic rate laws (lines 152 to 153, the reviewer assumed this refers to a "k_cat" with units of inverse time, so it is understood that "k_cat" = 1/"tau"). In enzyme kinetics, turnover number is a constant for the system (e.g. "k_cat" = "V_max" / "E_t"). So Table 1 and the beginning of the manuscript suggest a constant "tau" value. However, In line 148, this statement is made: "...and adding the kinetic rate laws 'tau' and density 'rho'". Further, in lines 156 to 158, it is stated that "tau" is dependent on concentrations for both participant and non-participant metabolites. In Eq. 4, τ is treated as a concentration-dependent function. In these lines and equations, it appears that "tau" evolves from a simple turnover time vector into a Michaelis-Menten form description of the reaction rate, as lines 161 to 165 discuss Michaelis constants and turnover numbers, as well as the unit for input to this equation, and line 333 discusses using a "tau" that follows "a simple Michaelis-Menten rate law".

Response: We now see that the similarity between the terms "turnover time" and "turnover number", may lead to confusion. The assumption that the "turnover time" tau is simply the inverse of the "turnover number" k_cat is plausible (although not stated in the text) but incorrect, and hence the text may be confusing but is not inconsistent. The relationship between tau and k_cat is explicitly shown in Fig. 1, and it is explained in detail in S2 Appendix, as indicated on line 170.

Action: We updated the sentence on line 169 to clarify this issue.

12) How are proteins included and formulated in the model? Is it then that there is a single pseudo-protein in the GBA models (the section from lines 118 to 120 is written in the singular form, indicating a single protein metabolite and single protein-producing reaction from the ribosome)? How is the composition of this single protein determined? Note that this seems to contradict lines 149 to 151, Eqs. 4 and 5, as well as many other places in the manuscript which suggest that each reaction j has an associated protein or protein complex.

Response: We see how our treatment of proteins might be confusing, and appreciate this important point. We do not consider a single protein, it is just useful mathematically to define a variable c^a for the sum of protein concentrations. Individual proteins have individual concentrations p^j. We assume that the ribosome produces individual proteins in proportion to their individual concentration to offset their dilution, at the rate μp^j. Mathematically, it is convenient to summarize the production of all proteins by setting the overall rate of protein production to μc^a. The amino acid composition of proteins – which is assumed to be identical for all proteins – is defined by the column "r" of M, which corresponds to the ribosome reaction.

Action: We clarified these issues by updating line 217.

13) This manuscript is considered by the reviewer to be incomplete because no (biologically) relevant examples and applications are provided for the methods and computational models. The authors mentioned examples in Supplementary Materials but they are only simple toy examples.

Response: We see how examples might help readers to understand the mathematical formulation, and how applications might clarify the importance of theoretical results. However, constructing a biologically relevant example is a substantial project in its own right and is well beyond the scope of the current manuscript. Moreover, we disagree that biologically relevant examples and applications are strictly necessary to make any study complete, as this would rule out any purely theoretical work in biology. Instead, we believe that such work is important, and the fact that such articles have been published in the past by this journal indicates that the editors generally agree with this notion (see, e.g., https://doi.org/10.1371/journal.pcbi.1007559).

Action: None.

14) If the tool requires the use of pre-defined kinetic law (in "tau") with pre-defined kinetic parameters, then some other tool would need to be used in conjunction with this modeling framework. This is acknowledged in Lines 592 to 595. However, many such parameter estimating tools or approaches (such as ensemble modeling) integrate parameter estimation with kinetic parameter estimation. Therefore, what would be the advantage of using this approach as opposed to an integrated approach? How do these model structures compare with other kinetic modeling approaches?

Response: We appreciate the problem of limited kinetic parameters, and indeed address this issue in line 648. However, in our view, formulating a modeling framework and constructing or parameterising a particular model are very distinct issues. In this work we provide some advancement on the first issue. We point out that in the mathematical theory of metabolic modeling, one example being Metabolic Control Analysis (MCA), it is customary to assume and prove theorems about a "linear metabolic chain" without specifying the kinetic parameters, nor indicating how exactly these should be estimated. We emphasize that, by design, our method holds for arbitrary rate laws; and since we do not restrict what rate laws should be used, it is in fact impossible to prescribe how the parameters should be estimated in general.

Action: None.

15) It appears that a number of bilinear terms exist in the formulation. How is this consistent with the purported convexity of the formulation?

Response: The question of convexity is indeed important for simulating models and finding optimal growth states. However, we do not state in the manuscript that the optimization is convex. We do not focus on methods to find optimal states, but instead focus on what the mathematical properties of these states are. We see how this differs from what is usually done in metabolic analysis.

Action: We now address the possible uniqueness of solutions on line 429.

Minor concerns

16) Line 15, Constraint Based Reconstruction and Analysis (COBRA) and Genome-Scale Model (GSM or GEM) are more commonly used acronyms, and should be included here for clarity and easier linking to related works.

Response: In our view CBM provides a more general terminology, and fits better the simpler overview intended in our introduction. We explicitly mention methods like FBA and RBA in the next paragraphs, and we link to the most important studies, so we believe that the text is sufficiently clear and easy to link to related works.

Action: None.

17) Lines 33-40, is the argument being made that RBA and ME models are both CBM models? If so, specify. Many other works consider these a different class of models from GSM models, so this could be unintentionally confusing if not clarified.

Response: RBA and ME models are also based on optimization under (essentially linear) constraints, so we indeed consider them to be CBM. However, we agree that this can potentially lead to confusion.

Action: We now clarify this issue on line 34.

18) Lines 43-44, "widely adopted" rather than "most powerful" should be used.

Action: Changed to “most efficient”, to avoid the repetition of "widely adopted".

19) Table 1, please make sure that a symbol is defined before it is used (index i is noted as containing m and a, but m is not yet defined).

Action: Table 1 is updated as suggested.

20) Line 110, for clarity please specify if M,τ, and ρ are parameters or variables.

Action: We updated line 122 to specify this as suggested.

21) Lines 123-124, please specify if the negative and positive entries are within a particular column.

Action: We updated line 137 to clarify that.

22) Line 163, please ensure consistency in how units are described. In Table 1, the units for v are described as "[mass][volume]^-1[time]^-1" whereas here they are described as "[mass x volume ^-1 x time ^-1]". This happens elsewhere as well, this example is intended to draw all such instances to the authors' attention.

Action: Corrected.

23) Line 168, could the value for "rho" be provided in the main text?

Response: The value of rho must be determined for a given particular model. As we do not present a particular model in the main text, we can also not provide a specific value for \rho.

Action: None.

24) Line 237, please specify the "the previous two equations".

Action: Corrected.

Reviewer #2

This is a well-written and most welcome paper on the conditions that hold in states of optimal balanced growth, in the particular case of an EFM/EGM, and in which it is assumed that all protein and ribosome in the cell may be lumped into one protein compartment. I particularly like the new view of reformulating the balanced growth equations using flux fractions, and the fact that in this particular model the metabolite concentration indeed drop out of the equations, even though they were explicitly taken into account to start with.

I have enjoyed reading the manuscript, and only have a number of comments for improvement, clarification and link to other literature.

Response: We are grateful for the careful and constructive review of our manuscript, and implemented a number of improvements in response, as detailed below.

1) One of the omissions that I think should really be dealt with is the link to the elementary mode literature. The case considered is that in which the reaction matrix has full rank. This is equivalent to restricting to an Elementary Mode, whether it is an Elem. Flux Mode [refs 21 ,22 in the ms] or an Elem Growth Mode [ref 44,45, maybe also 46?], as I am sure the authors are well aware. Given the central role E(F/G)Ms have come to play in our systems biology

literature, I think it is essential that this concept is mentioned, rather than just refer to papers that deal with them.

Response: The central mathematical result of our study is the derivation of the (necessary) analytical conditions for GBA models with arbitrary matrix **M**, generalizing our previous analytical study restricted to GBA models with full column rank **M** (Dourado & Lercher 2020). The analytical conditions derived here represent a novel result, presenting the exact condition that determines whether a given reaction is active or not in the optimal growth state (OGS), now defined explicitly. Thus, the connection to the whole body of work on EFMs and EGMs is indeed very relevant; however, this background is not absolutely necessary to follow our other results in this study. Because our manuscript is already quite dense with mathematical details and interpretations, and considering the allowed page limit, we now explain these connections more clearly but leave a more detailed discussion for future studies.

Action: We added this discussion on line 202 to clarify the connection to EGMs and EFMs.

2) In l 87, the authors refer to two papers in which it is proved that EFMs are specific flux optimisers. In [44], it is shown that EGMs are growth rate optimisers. The model considered in this paper falls somewhat in the middle of these approaches (EFMs disregarding the self-replication aspect and not accounting for protein synthesis, EGMs accounting for everything, differentiating between different enzyme synthesis rates, etc.), and it is not immediately clear to me that the EFM proofs apply to the case at hand. Maybe it is best to refer to [44] (and maybe also [45] which deals with other cases in-between EFMs and EGMs and also contains proofs of growth rate optimisation in elementary modes) when making the claim that one may restrict to the case of full matrix rank. Then all bases are covered.

Response: As outlined in the previous answer, in this study we do not need to assume that the matrix **M** is of full column rank. Nevertheless, in our previous study (Dourado & Lercher, 2020; Ref. [4]), we provided a mathematical proof that the optimal states of GBA models (OGS) are also minimal sets of active reactions (we term these Elementary Growth States, indeed not exactly EFM nor EGM) with full column rank **M**, formally generalizing the previous results in Refs. [21], [22].

Action: The added discussion on line 202 clarifies these points.

3) l 117 and further: Here the matrix M is introduced. I had trouble understanding the construction of the last row of M, even though it turned out to be trivial. I do not think the construction follows Molenaar et al (who did differentiate between protein compartments, and were the inspiration of the introduction of the 'alpha' ribosomal fractions used in [44]). Please explain this last row more clearly, preferably by giving a tiny example. At present, examples are in the SI (but this is not mentioned in the ms at this point, so this could be another solution), which is not too accessible to the reader.

Response: We indeed simplified the treatment of Molenaar et al. by lumping all protein together – essentially assuming identical composition and translation efficiency for all proteins. This greatly simplifies the analytical treatment, we believe at only a minor cost in terms of accuracy. We agree with the Reviewer that this difference needs to be stated more clearly.

Action: We added more explanation on line 131 to clarify this issue.

4) In Eq (5) - (6) there are two constraints, but in l 192 it is mentioned that the protein constraint is an emergent property, rather than a real hard constraint (i.e. c^a is not known beforehand). So why include it then? I guess I'm missing something here.

Response: Equation (5) simply states that the individual protein mass concentrations must sum to c^a. In our framework, this total protein mass concentration is a variable, it is not assumed to be known or constrained. c^a is, of course, constrained by the total density via Eq. (6); moreover, it is used to mathematically describe the mass conservation for proteins via the corresponding row "a" in Eq. (3).

Action: To make these issues explicit in the manuscript, we added an explanation on line 217.

5) l 242: the recursion has been noted in older papers, starting with the RBA models by Goelzer et al (2009). It is a mainstay of ME-type models. It is clearly also mentioned in [44]. The insight that this recursion disappears here is very neat, and I need to think about that more deeply. But please add some refs here.

Response: We thank the Reviewer for pointing out this omission.

Action: We added a reference to [Goelzer et al, deGroot et al.], as suggested.

6) Growth analysis, page 9.

   This whole section aims to derive conditions that hold at optimality + steady state. In particular for EFMs (without the self-replication part taken into account), this has been done in Planqué et al. (2018), in which such equations are coupled to dynamic enzyme synthesis rates to control the maximal specific flux in varying environments. The situation here is of course a bit different, but the two situations are closely related, so a reference seems in place, either here or in the Discussion.

Response: We thank the Reviewer for making us aware of this reference. The analytical conditions for EFM at steady-state without self-replication (and without metabolite dilution by growth) were also presented in 1991 by Klipp & Heinrich, which is already cited.

Action: We added the Planqué et al. (2018) citation in the Discussion of a possible extension to optimal dynamical states.

7) In this section, it is also not mentioned whether such optimal states actually necessarily exist, or whether there are multiple (local) optima. This all has to do with the convexity properties of the relevant functional, of which the authors are well aware. References such as the paper by Wolfram and Elad in 2016 on Enzyme Cost Minimisation and convexity, and also (Planqué et al. 2018) which improves this slightly to strict convexity, are relevant, but I don't think they solve the case here immediately. The authors would do well to change 'the optimal state' to 'an optimal state' in several places, such as in line 345 and 385.

Response: The issue of convexity is in fact not essential in our present discussion, and we made the conscious choice of leaving it out to not complicate the mathematical treatment further. In this study, we are interested in the properties of the global optimum, and it is in fact not a problem that the same equations also hold for other local optima (if they exist).

Action: We updated line 429 to clarify that.

Discussion

8) l 606: I read there are local conditions for each reaction as necessary conditions for optimal growth. I didn't quite understand this part of the ms, I have to say (I didn't have the time to think in detail about it), but I find this surprising. Surely, because reactions have substrates and products, such conditions must be coupled? See Planqué et al (2018) for a situation where this is clearly the case. But as I said, the situations do not exactly compare.

Response: Our use of the term “local” on line 606 is indeed confusing, especially because it’s not used with the same meaning as when introduced first on line 476, when referring to how Eq. 44 depends only on the local knowledge of the reaction network. We now restrict the use of “local” to the first case only.

Action: We updated line 660 to clarify that.

9) I somehow find the idea of lumping all protein and ribosome into one pool, while on the other hand calculating (differing) steady state protein and ribosome concentrations for different conditions peculiar. Would it be not more natural to explain this by saying that all protein synthesis rates (per unit of ribosome) are assumed to be equal (which is also what comes out of having a constant relevant amino acid abundance assumption, see l 638 and which is also discussed in [44])? Now it sometimes reads as if you both lump things, and not lump things. The idea of marginal protein allocation clearly hinges on proteins being present in different concentrations (in optimum or otherwise). I think this just needs to be clarified, preferably already in the part where the model is introduced.

Response: We see how our treatment of proteins might be confusing, and appreciate this important point.

Action: We clarified our assumptions and formulations, now stating explicitly that we consider all individual proteins as catalysts, but can lump the production of their combined mass because we assume identical amino acid compositions and identical translation efficiencies. Specifically, we updated line 217 to clarify that.

10) l 683: The situation considered here is that of optimal control, but there are alternatives, such as adaptive control. In the latter case, this is essentially the qORAC like framework, see Planqué et al (2018). As it already exists (for a slightly different case than the one considered here), and the extension from qORAC to the present paper would be only a small change (I think), it should be cited here.

Response: qORAC is indeed related to the growth problem described here. Extending qORAC to the GBA framework is not trivial even in the adaptive control scenario (i.e., growth rate assumed to be optimal at each time point), for the same main reasons that hamper the extensions of other long established results in MCA. These reasons are based on the following assumptions of qORAC and related work: i) the assumption that an "output flux" at fixed invested enzyme must be maximized (or equivalently, invested enzyme must be minimized for a fixed output flux), which is not the same as the explicit maximization of growth rate under the constraints described here; ii) the assumption that the dilution by growth is negligible for metabolic intermediates; and iii) the restriction that the pathway must be an EFM. An analytical theory that overcomes the limitations inherent in these assumptions is indeed the main accomplishment of our presented GBA framework extension, and we do not see how a small change in the qORAC framework would achieve the same results – achieving the same results would require to rederive everything from first principles in the same manner as presented here. However, we do agree that the qORAC framework provides a pointer to possible future developments.

Action: We now refer to the qORAC framework in the Discussion (line 742).

Minor comments and questions:

11) l 36: the burden => the enzyme/protein burden?

Response: The full sentence reads "In contrast to FBA, these methods take into account the burden of producing the macromolecules required for catalyzing each flux". We use "macromolecules" here because the burden is not only proteins, but also other molecules such as RNA.

Action: We updated line 38 to clarify this issue.

12) l 72: what is the difference between a fixed protein concentration and a fixed combined mass density of their components? I think I understand because I know how this is usually formulated, but the reader might not.

Response: The sentence was indeed not clear.

Action: We update line 72 to clarify this sentence.

13) l 284: set OF kinetic parameters

Action: Indeed, corrected.

14) l 294: What are shadow prices? I understand there must be some link to economic arguments, but this needs to be explained, or at least given some reference to aid the reader.

Action: We added an explanation (line 344) and a reference as suggested [Liebermeister W. (2022) bioRxiv doi:10.1101/483891].

References:

Planqué et al. (2018). Maintaining maximal metabolic rates by gene expression control. PLoS Comp Biol. 14(9):e1006412.

Reviewer #3: [Elad Noor]

In this work, Dourado et al. extend their framework called Growth Balance Analysis to a more general system with several meaningful advantages. All formulae are expressed using a single independent variable f (for flux) and therefore are simpler than other types of cell growth models. By this, they lay the groundwork for a universal modeling approach which has the potential to bring together many disjoint approaches and hopefully increase cooperation between modelers that use FBA, MCA, kinetic models and others. The text is very well written and I haven't found any scientific or mathematical issues.

I can offer a few suggestions that might improve the text even further:

1. The use of Einstein's summation convention is, in my view, not very helpful. Indeed, it might appeal to some physicists and could be a bit less verbose - but I don't think the benefit outweighs the downside of being less standard and harder to read for many people.

Response: We have to agree with the reviewer.

Action: We updated the notation to the usual matrix/vector multiplication.

2. I might have misunderstood, but it appears that fluxes (v^j) and turnover times (τ^j) can be negative (and indeed they are not constrained to be non-negative like other variables). I think stating this explicitly could help readability.

Response: Agreed.

Action: We updated the sentence on line 168, and added an explanation on line 326.

3. At some point, metabolite concentrations and protein concentrations are "replaced" with fluxes based on the balanced growth assumption (i.e. unlike in FBA where fluxes balance to 0, here each one balances to the dilution rate of the metabolite/protein defined by cellular growth). First, this idea was presented in a similar context in 2010 by Benyamini et al. (https://doi.org/10.1186/gb-2010-11-4-r43), albeit only for metabolites.

Response: We are grateful for the reference. The MD-FBA framework does account for a single fixed lower bound on the dilution of each active metabolite, providing more realistic predictions than FBA. It however cannot capture how different metabolites have different concentrations, and does not replace metabolite concentrations with fluxes in the same way done in this study.

Action: We now cite Benyamini et al. on line 217.

Furthermore, in the discussion (lines 602-603) the authors highlight the advantage of using this approach in minimizing the number of independent variables and thus assisting the numerical solvers. However, this might be a slight overstatement since many models indeed have explicit protein concentration variables, but then the biosynthesis flux is the dependent variable (and very simple to express as a function of the concentration). For metabolites, the case is not very different (the Sv = 0 constraint might have more rows and be more rank deficient, but almost all solvers can deal with this easily).

Response: The search space associated with our problem formulation in terms of scaled fluxes **f** has a lower dimension than the space associated with metabolite and protein concentrations, and we thus expect that this formulation will support faster numerical solutions. However, we agree that without experiments on diverse example models, we cannot demonstrate such an advantage.

Action: We have toned down the formulation as suggested and updated line 657.

4. The manuscript is quite long and dense (in terms of mathematical definitions and derivations). There is no easy solution to this, but perhaps the authors might consider splitting it or moving some parts to a supplementary section and really focus only on the main message. In addition, perhaps a few toy examples (with simulations or analytical solutions) could be helpful as keeping track of all the abstract math symbols all the way until the end is a bit daunting. That being said, I greatly appreciated the table of symbols on page 4. Perhaps one could add more of these symbols to figure 1A as well?

Response: We put a lot of effort into deciding what should be in the main text and what can be left out or delegated to the SI, and we feel that the current version is close to optimal in terms of the message that we want to convey. We do, however, agree that the manuscript is very dense. We also agree that some examples would provide more context, but the page limit pressures us to leave these also in the SI.

Action: We moved the detailed mathematical derivations of Eqs. (26)-(30) and (33)-(35) to the Methods section.
